# Fenchone Derivatives as a Novel Class of CB2 Selective Ligands: Design, Synthesis, X-ray Structure and Therapeutic Potential

**DOI:** 10.3390/molecules27041382

**Published:** 2022-02-18

**Authors:** Reem Smoum, Christeene Haj, Shira Hirsch, Alina Nemirovski, Zhannah Yekhtin, Benny Bogoslavsky, Gaganjyot Kaur Bakshi, Mukesh Chourasia, Ruth Gallily, Joseph Tam, Raphael Mechoulam

**Affiliations:** 1 The Institute for Drug Research, School of Pharmacy, Faculty of Medicine, The Hebrew University of Jerusalem, Jerusalem 9112102, Israel; christeeneh@ekmd.huji.ac.il (C.H.); shirah@ekmd.huji.ac.il (S.H.); alina.nemirovskai@mail.huji.ac.il (A.N.); yossi.tam@mail.huji.ac.il (J.T.); raphaelm@ekmd.huji.ac.il (R.M.); 2 The Lautenberg Center for Immunology and Cancer Research, Faculty of Medicine, The Hebrew University of Jerusalem, Jerusalem 9112102, Israel; zhannay@hadassah.org.il (Z.Y.); ruthg@ekmd.huji.ac.il (R.G.); 3 The Laboratory for Molecular Structure Analysis, The Institute of Chemistry, The Hebrew University of Jerusalem, Jerusalem 9190401, Israel; bennybo@savion.huji.ac.il; 4 Center for Computational Biology and Bioinformatics, Amity Institute of Biotechnology, Amity University Uttar Pradesh, Noida 201301, India; gaganjyot.bakshi@student.amity.edu (G.K.B.); mchourasia@gmail.com (M.C.)

**Keywords:** cannabinoid agonists, hCB2 receptor, inflammation, fenchone

## Abstract

A series of novel cannabinoid-type derivatives were synthesized by the coupling of (1S,4R)-(+) and (1R,4S)-(−)-fenchones with various resorcinols/phenols. The fenchone-resorcinol derivatives were fluorinated using Selectfluor and demethylated using sodium ethanethiolate in dimethylformamide (DMF). The absolute configurations of four compounds were determined by X-ray single crystal diffraction. The fenchone-resorcinol analogs possessed high affinity and selectivity for the CB2 cannabinoid receptor. One of the analogues synthesized, 2-(2′,6′-dimethoxy-4′-(2″-methyloctan-2″-yl)phenyl)-1,3,3-trimethylbicyclo[2.2.1]heptan-2-ol (**1d**), had a high affinity (K_i_ = 3.51 nM) and selectivity for the human CB2 receptor (hCB2). In the [^35^S]GTPγS binding assay, our lead compound was found to be a highly potent and efficacious hCB2 receptor agonist (EC_50_ = 2.59 nM, E_(max)_ = 89.6%). Two of the fenchone derivatives were found to possess anti-inflammatory and analgesic properties. Molecular-modeling studies elucidated the binding interactions of **1d** within the CB2 binding site.

## 1. Introduction

Fenchone is a bicyclic monoterpene present in essential oils of plant species [1] and is a component of the volatile oil of fresh and air-dried buds of *Cannabis sativa* [2]. It exerts anti-inflammatory action in rats as noted in a carrageenan-induced right hind-paw edema model [3]. Being a major constituent of *Foeniculum vulgare* essential oil, fenchone was shown to have an anti-nociceptive activity in the tail-flick pain mouse model, without inducing motor incoordination [4]. Recent data have demonstrated the protective effects of *Lavandula stoechas* essential oil, where the principal compound is D-fenchone (29.28%), against diabetes and oxidative stress induced by alloxan treatment in rats. Lavender essential oils also decrease kidney and hepatic injuries through their antioxidant properties and play a major role as hepato- and nephroprotection products [5].

Monoterpenes and 5-substituted resorcinols are widely used for the syntheses of cannabinoids [6]. Many of them modulate the endocannabinoid system (ECS), which is an emerging target for the regulation of inflammation and the immune response [7]. ECS activation occurs either via ligands binding to the cannabinoid receptors 1 (CB1R) and 2 (CB2R) or in an indirect way, by promoting the synthesis of endocannabinoids, or, alternatively, in inhibiting their degradation. CB1R is abundant in the central nervous system (CNS) and mediates the classical psychotropic effects, whereas CB2R is mainly expressed in the cells and tissues of the immune system and the astrocytes and microglia in the CNS [8].

**Figure 1:**
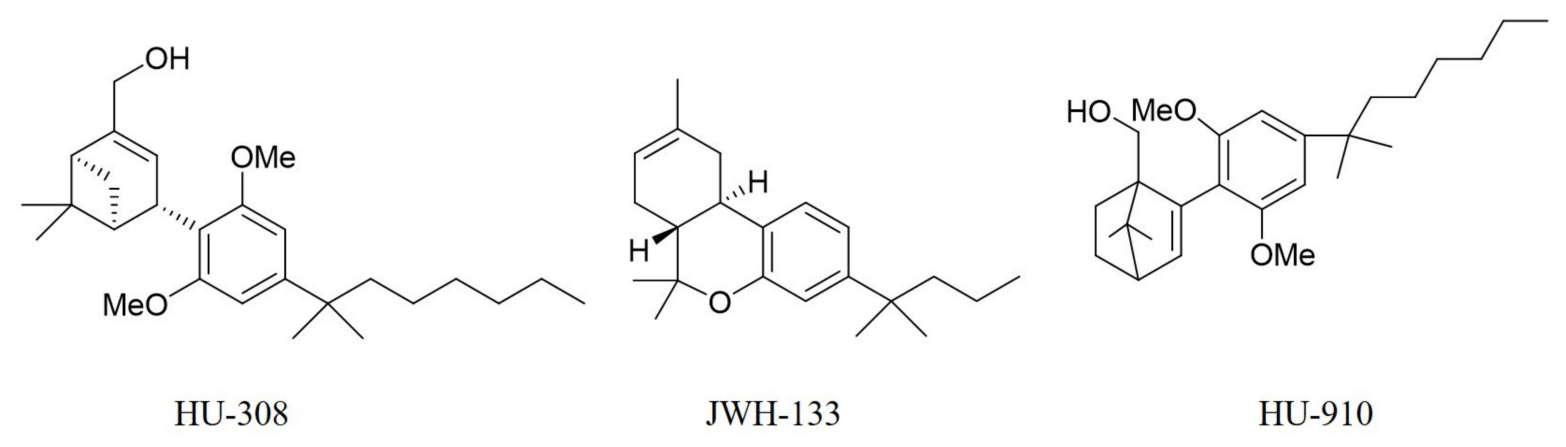
Chemical structure of some CB2 receptor agonists.

CB2R has become an attractive target since it does not cause the adverse psychotropic effects associated with CB1R activation. Activation of CB2R inhibits upstream and downstream molecules of the inflammatory process, and its stimulation exerts analgesic activity. It is known to be up-regulated in pathological conditions correlated with the onset of inflammatory events in cancer and neurodegenerative diseases [9]. CB2 agonists restrain inflammatory responses in hepatic ischemia-reperfusion injury [10], uveitis [11] and contact dermatitis [12]. Some synthetic agonists, such as HU-308 [12], JWH-133 [13] and HU-910 [14] (Figure 1) have terpene and resorcinol-derived moieties in their structure, and hence they resemble the phytocannabinoids Δ^9^-tetrahydrocannabinol (Δ^9^-THC) and cannabidiol (CBD). Other, non-phytocannabinoid-type agonists have also been reported [15].

**Figure 2:**
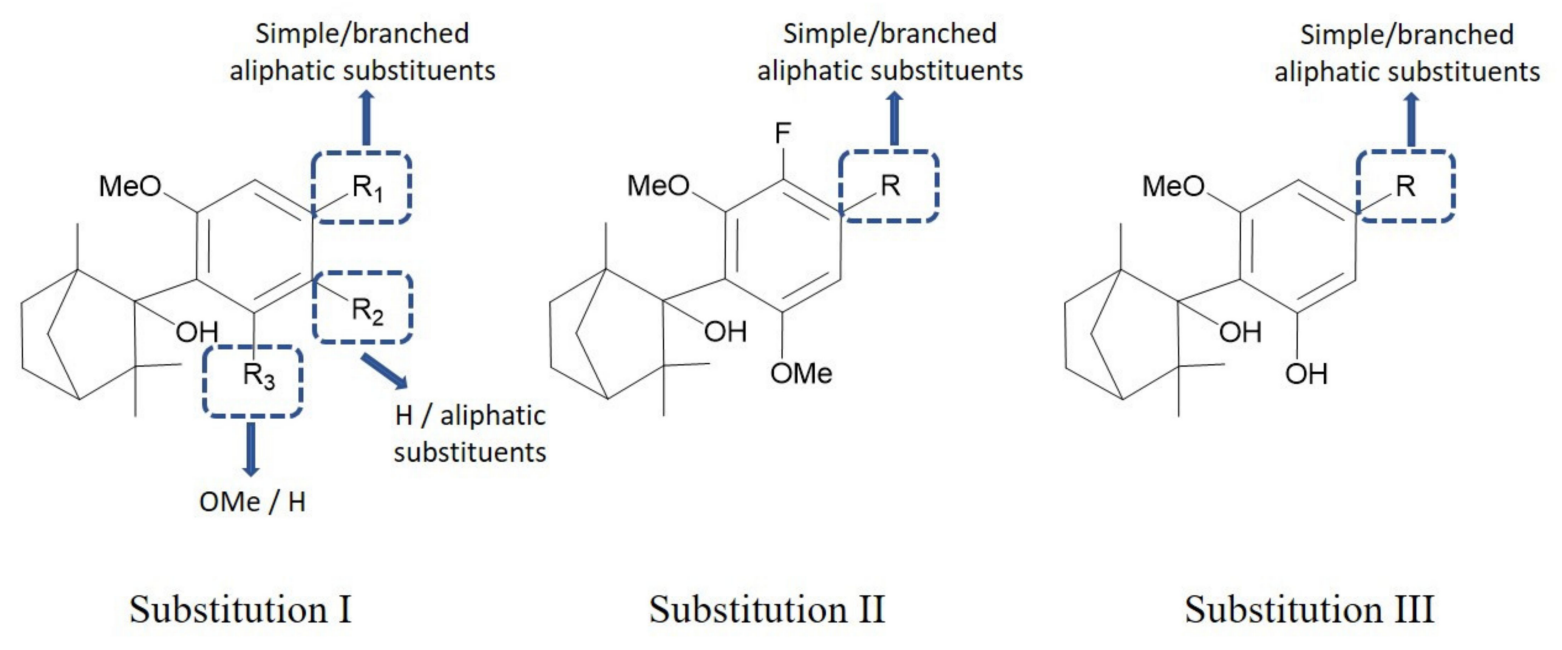
Structural modifications employed in the SAR study.

Here, we present the synthesis and structural identification of twenty-four novel bicyclic monoterpenoid fenchone derivatives with different alkylresorcinol and alkylphenyl groups. We started with the synthesis of fenchone-alkylresorcinols and fenchone-alkylphenols (Figure 2, Substitution I) in alignment with the previously reported HU-308 [12], JWH-133 [13] and HU-910 [14] (Figure 1). Next, we explored the effect of fluorination of the aromatic ring in the fenchone-alkylresorcinols (Figure 2, Substitution II) bearing different aliphatic substituents. Then, the fenchone-alkylresorcinols with different alkyl substituents were demethylated (Figure 2, Substitution III).

The compounds were characterized by NMR, GCMS and LC-UV-MS (ESI). 1D and 2D NMR experiments (DEPT, gCOSY, TOCSY, HSQC and HMBC) were used to determine the structure assignment of three different fenchone derivatives. The absolute configurations of four derivatives were determined by single-crystal X-ray diffraction. The binding affinities of the fenchone derivatives at the human CB1R (hCB1R) and CB2R (hCB2R) were assessed. Affinity data (K_i_ values) were used to calculate the selectivity indices of these compounds. These ligands were also examined in the [^35^S]GTPγS binding assay with the aim of evaluation of their functional activity. To assess the in vivo efficacy of the newly developed chemotypes, two compounds from the most potent series were selected to be tested for their anti-inflammatory and anti-nociceptive properties. In addition, we carried out molecular-modeling studies to know the binding interactions of **1d** within the CB2 binding site and compare with the parent CB2 compounds.

## 2. Results and Discussion

### 2.1. Chemistry

**Figure 3:**
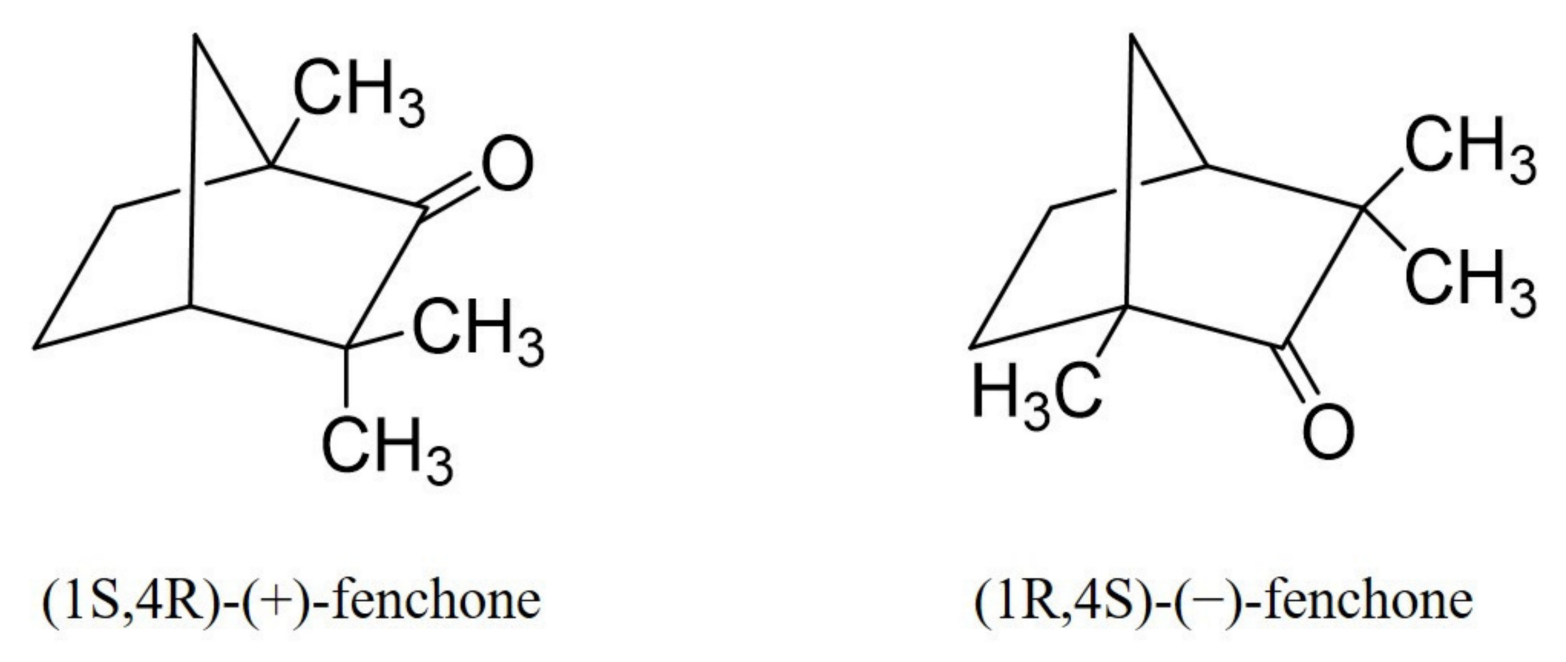
Chemical structures of (1S,4R)-(+)-fenchone and (1R,4S)-(−)-fenchone.

**Scheme 1:**
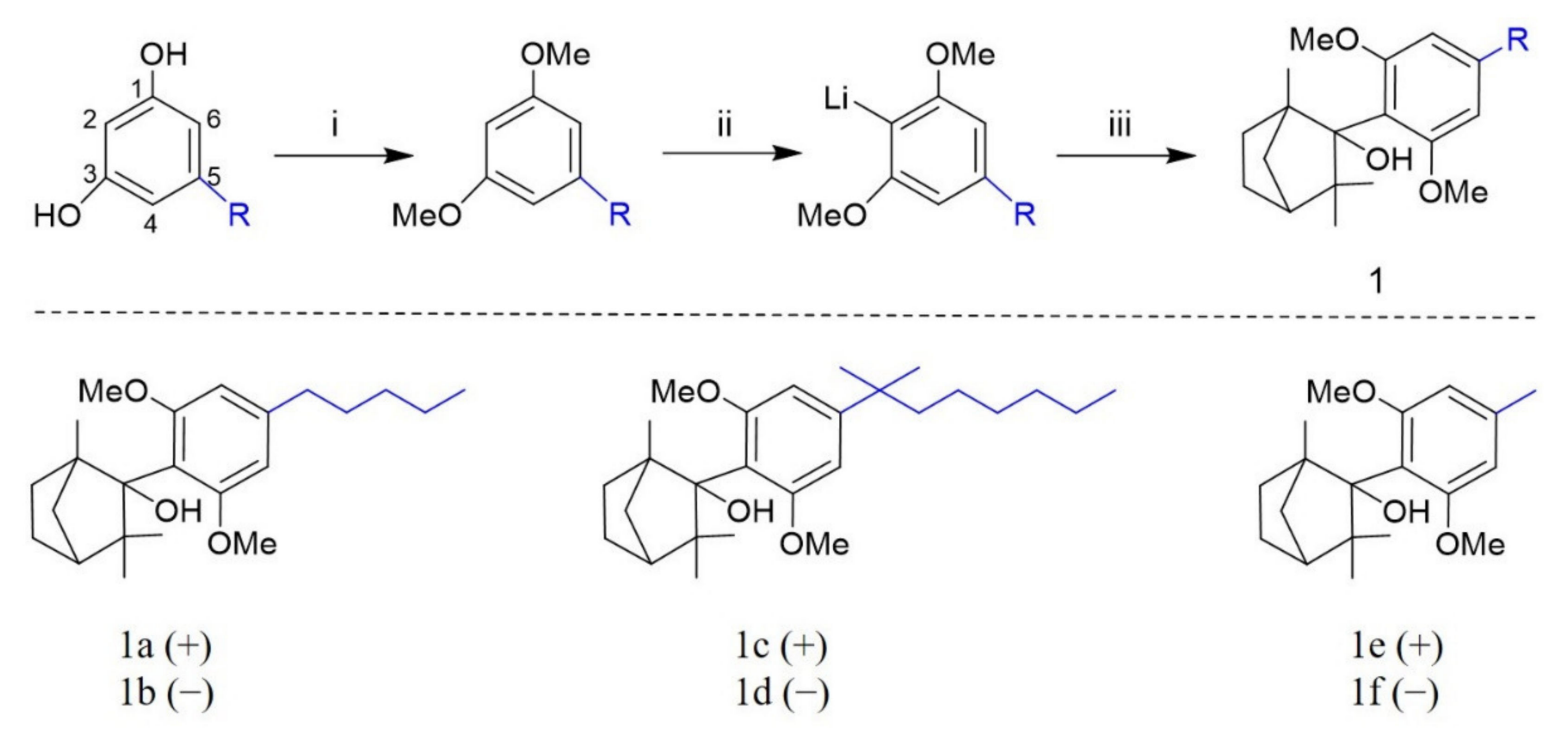
Synthesis of 2-(2′,6′-dimethoxy-4′-alkylphenyl)-1,3,3-trimethylbicyclo[2.2.1]heptan-2-ol analogs (**1a**–**1f**) ^a^. ^a^ Reagents and conditions: (i) Potassium carbonate, anhydrous DMF and iodomethane. (ii) n-Butyllithium in hexane and anhydrous THF. (iii) Fenchone and anhydrous THF.

**Scheme 2:**
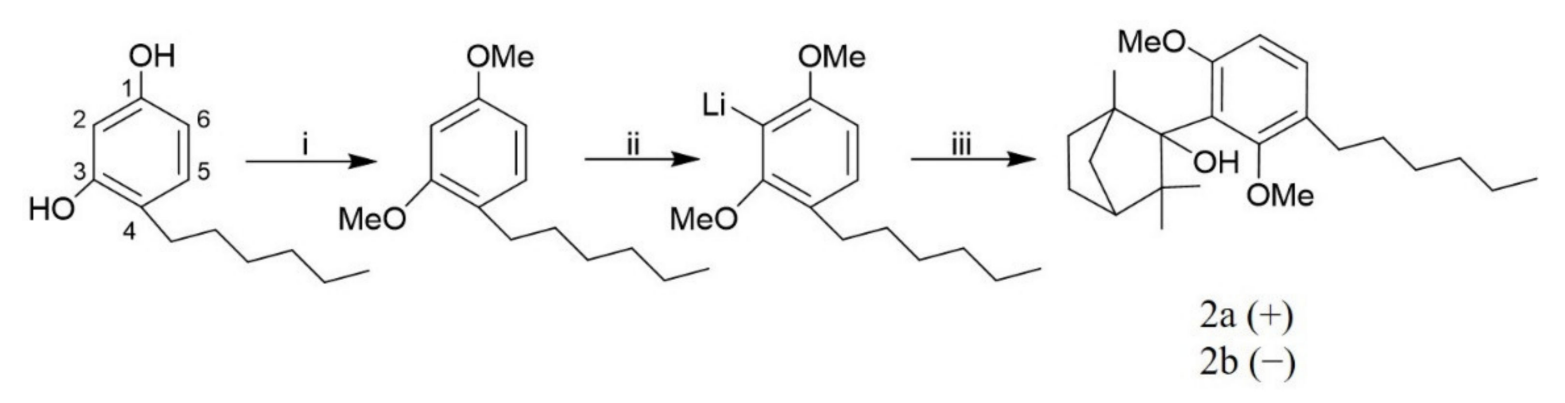
Synthesis of 2-(3′-hexyl-2′,6′-dimethoxyphenyl)-1,3,3-trimethylbicyclo[2.2.1]heptan-2-ol analogs (**2a**, **2b**) ^a^. ^a^ Reagents and conditions: (i) Potassium carbonate, anhydrous DMF and iodomethane. (ii) n-Butyllithium in hexane and anhydrous THF. (iii) Fenchone and anhydrous THF.

**Scheme 3:**
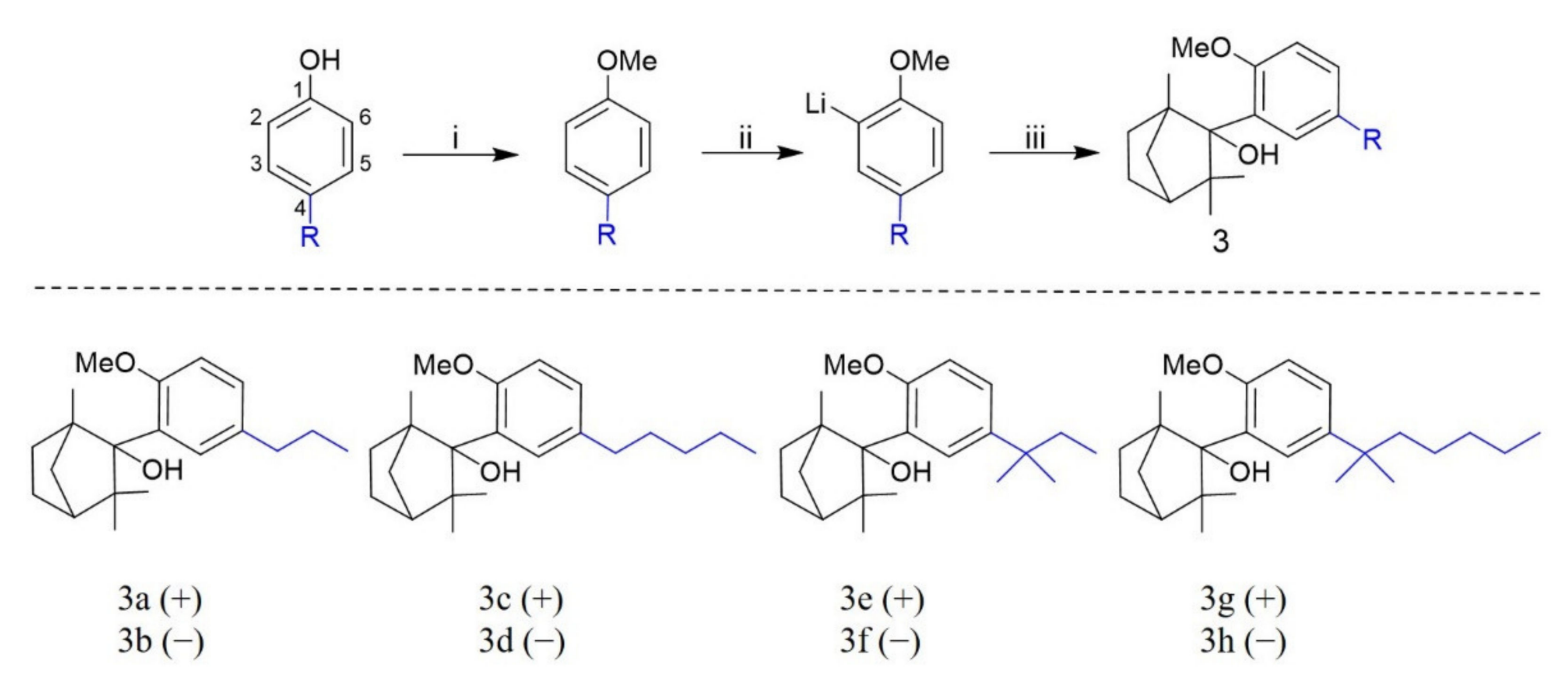
Synthesis of 2-(2′-methoxy-5′-alkylphenyl)-1,3,3-trimethylbicyclo[2.2.1]heptan-2-ol analogs (**3a**–**3h**) ^a^. ^a^ Reagents and conditions: (i) Potassium carbonate, anhydrous DMF and iodomethane. (ii) n-Butyllithium in hexane and anhydrous THF. (iii) Fenchone and anhydrous THF.

**Scheme 4:**
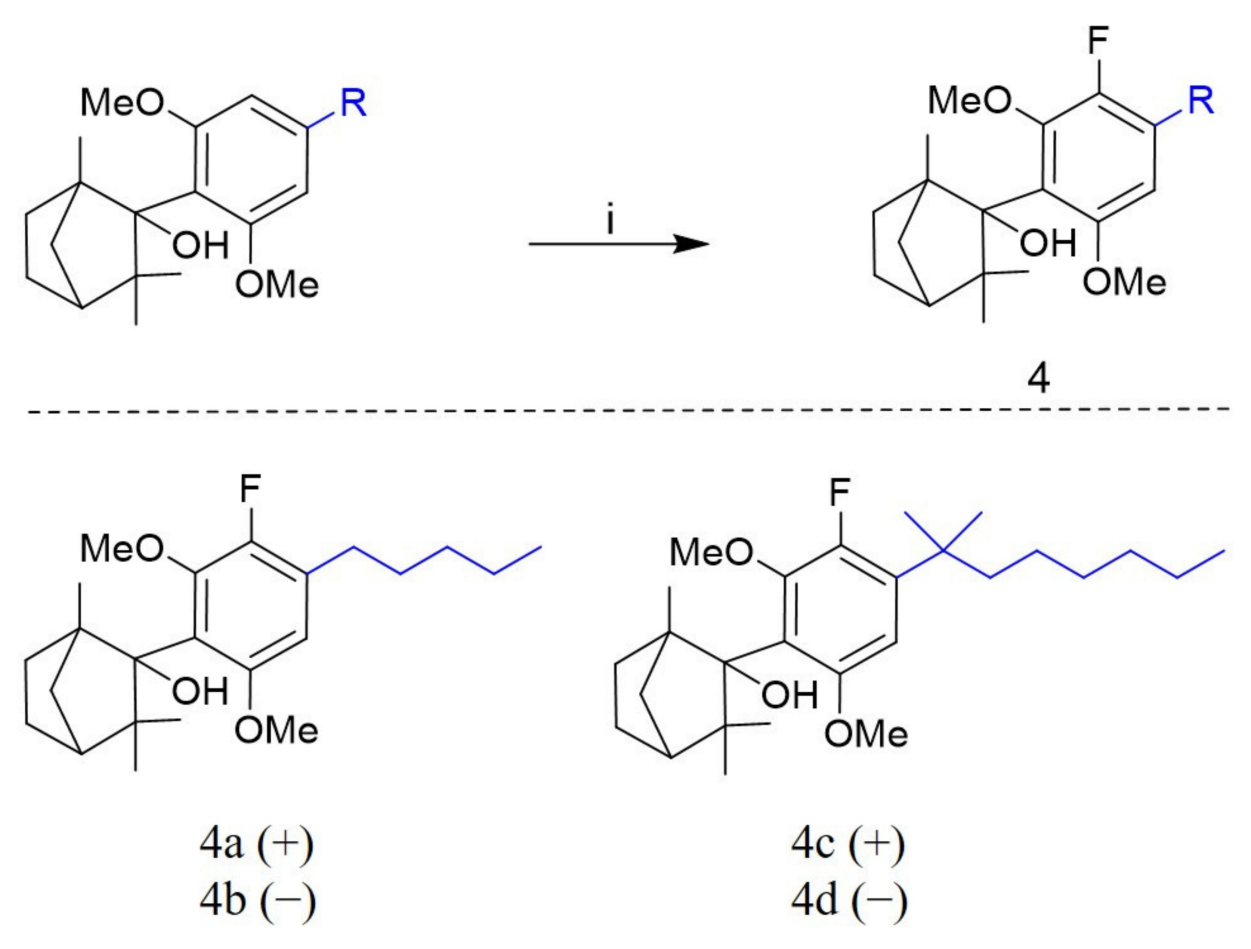
Synthesis of 2-(3′-fluoro-2′,6′-dimethoxy-4′-alkylphenyl)-1,3,3-trimethylbicyclo [2.2.1]heptan-2-ol analogs (**4a**–**4d**) ^a^. ^a^ Reagents and conditions: (i) Selectfluor and CH_3_CN.

(1S,4R)-(+)-fenchone and (1R,4S)-(−)-fenchone (Figure 3) were used in this study to synthesize novel cannabinoid-like compounds. The general routes to the synthesis of the targeted fenchone-resorcinol/phenol are shown in Scheme 1, Scheme 2 and Scheme 3. Introducing a 2-aryl substituent onto the fenchyl system has been previously reported [16,17]. The synthesis is mainly comprised of a three-step sequence. In the first step, alkylresorcinol dimethyl ethers/alkylphenol methyl ethers were prepared in good yields from the corresponding 5-alkyl resorcinols (olivetol, 5-(1,1-dimethylheptyl)resorcinol and orcinol) (Scheme 1), 4-hexyl resorcinol (Scheme 2) and 4-alkylphenols (4-propylphenol, 4-pentylphenol, 4-*tert*-amylphenol and 4-tert-octylphenol) (Scheme 3) using potassium carbonate, anhydrous DMF and iodomethane. The 2-lithio derivatives of the corresponding alkylresorcinol dimethyl ether/alkylphenol methyl ether were then prepared using n-butyllithium/hexane in anhydrous tetrahydrofuran (THF). The final step was the condensation with the (+/−)-fenchone to give the final products (Scheme 1 for 1,3-dimethoxy-5-alkylbenzene; Scheme 2 for 1-hexyl-2,4-dimethoxybenzene; and Scheme 3 for 1-methoxy-4-alkylbenzene).

Different resorcinols/phenols required different temperatures and reaction times for lithiation and condensation. The (1S,4R)-(+)-fenchone led to (+)-fenchone-resorcinol/-phenol products, while the (1R,4S)-(−)-fenchone led to (−)-fenchone-resorcinol/phenol products. The synthetic pathway from fenchone to the derivatives is short, and the yields vary from 20–62% depending on the resorcinol/phenol used.

Introducing fluorine into such molecules can affect conformation, pKa, intrinsic potency, membrane permeability, metabolic pathways and pharmacokinetic properties [18]. Thereby, fluorinated compounds at the aromatic ring of the fenchone derivatives (**1a**–**d**) were synthesized (Scheme 4). They were obtained by the reaction of the fenchone-alkylresorcinol dimethyl ether derivatives with 1-chloromethyl-4-fluoro-1,4-diazonia-bicyclo [2.2.2]octane bis(tetra-fluoroborate) or Selectfluor [19].

The optimal conditions involved the use of the Selectfluor reagent in CH_3_CN at r.t under a nitrogen atmosphere. Selectfluor is one of the most reactive electrophilic fluorination reagents and is safe, nontoxic and easy to handle [20]. However, Selectfluor only worked with the fenchone-alkylresorcinol dimethyl ether derivatives (**1a**–**d**) with low yield. Fluorination of the fenchone derivatives with the alkylphenol methyl ether (**3a**–**h**) was not successful.

**Scheme 5:**
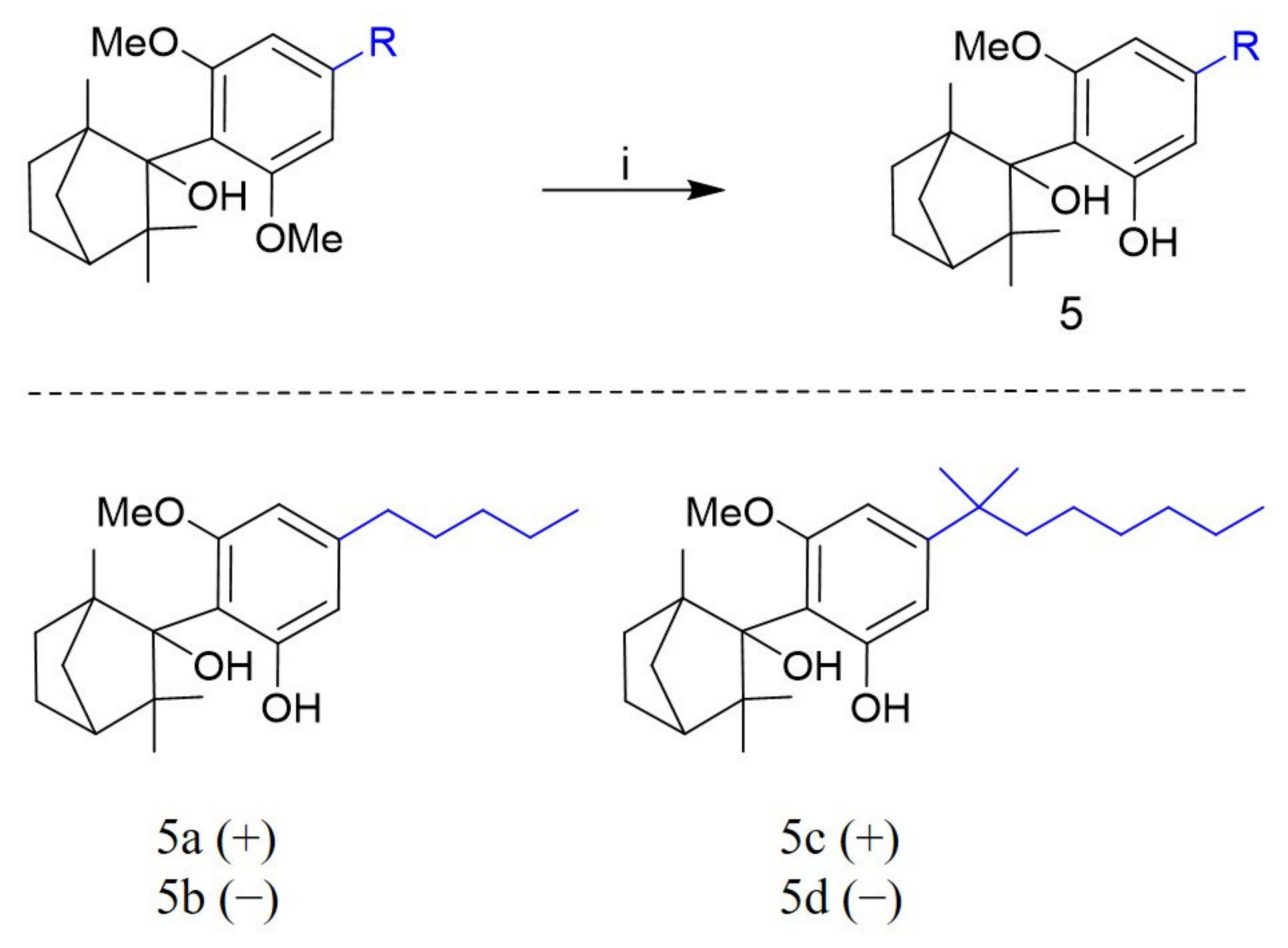
Synthesis of 2-(2′-hydroxy-6′-methoxy-4′-alkylphenyl)-1,3,3-trimethylbicyclo[2.2.1] heptan-2-ol (**5a**–**5d**) ^a^. ^a^ Reagents and conditions: (i) Sodium ethanethiolate and anhydrous DMF.

Demethylation of aromatic compounds involves the use of acids; however, fenchone upon treatment with acids undergoes rearrangement [21]. Therefore, the fenchone-alkylresorcinol dimethyl ether derivatives (**1a**–**d**) were demethylated with sodium ethanethiolate in DMF. However, only one methoxyl group was demethylated (Scheme 5) [22], and the yield was low. Moreover, when trying to demethylate the fenchone-alkylphenol methyl ether derivatives (**3a**–**h**), no reaction occurred, meaning that, in our series of compounds, this reagent works only with dimethoxy derivatives.

In total, 24 novel fenchone-based compounds were synthesized, and we grouped these in such a manner as to illustrate the effects of systematic structural variation.

### 2.2. NMR Analysis

The structures of all compounds were determined by ^1^H and ^13^C NMR and for the fluorinated compounds, ^19^F NMR was done. However, a complete analysis of 1D and 2D NMR spectra was performed for **1b, 1d and 5a**. The structures were assigned based on the analysis of ^1^H, ^13^C, DEPT, gCOSY, TOCSY, HSQC and HMBC NMR [23]. Through NMR analysis, it was possible to determine all the chemical shifts for all the carbons and hydrogens. The 2D HSQC permits to obtain a 2D heteronuclear chemical shift correlation map between directly-bonded ^1^H and X-heteronuclei (commonly, ^13^C and ^15^N). Following a ^1^H-^13^C-HSQC experiment for **1d** (Figure S1, Supplementary Materials), we saw that Carbon 2 (of the fenchone), connected to a hydroxyl group, did not have any cross peaks with the hydrogens and was shifted downfield (Supplementary Materials).

### 2.3. Description of the Crystal Structures

The crystals of **1b**, **1d**, **4b** and **5d** were prepared and determined by single crystal X-ray diffraction. Their crystal data and structure refinement are shown in Table S1. The observed hydrogen bonds are listed in Table S2. The molecular ellipsoids (at 50% probability) are shown in Figures S2 and S3.

### 2.4. Affinity for Cannabinoid Receptors

The compounds were further characterized using a radioligand binding assay to determine their affinities for CB1R and CB2R based on each test compound’s ability to displace the radiolabeled CB1R/CB2R agonist CP-55,940 from membranes of cells expressing the human CB1R and CB2R [24]. Inhibition constant values (K_i_) from the respective competition binding curves are listed in Table 1 in which **HU-308** was included for comparison.

**Figure 4:**
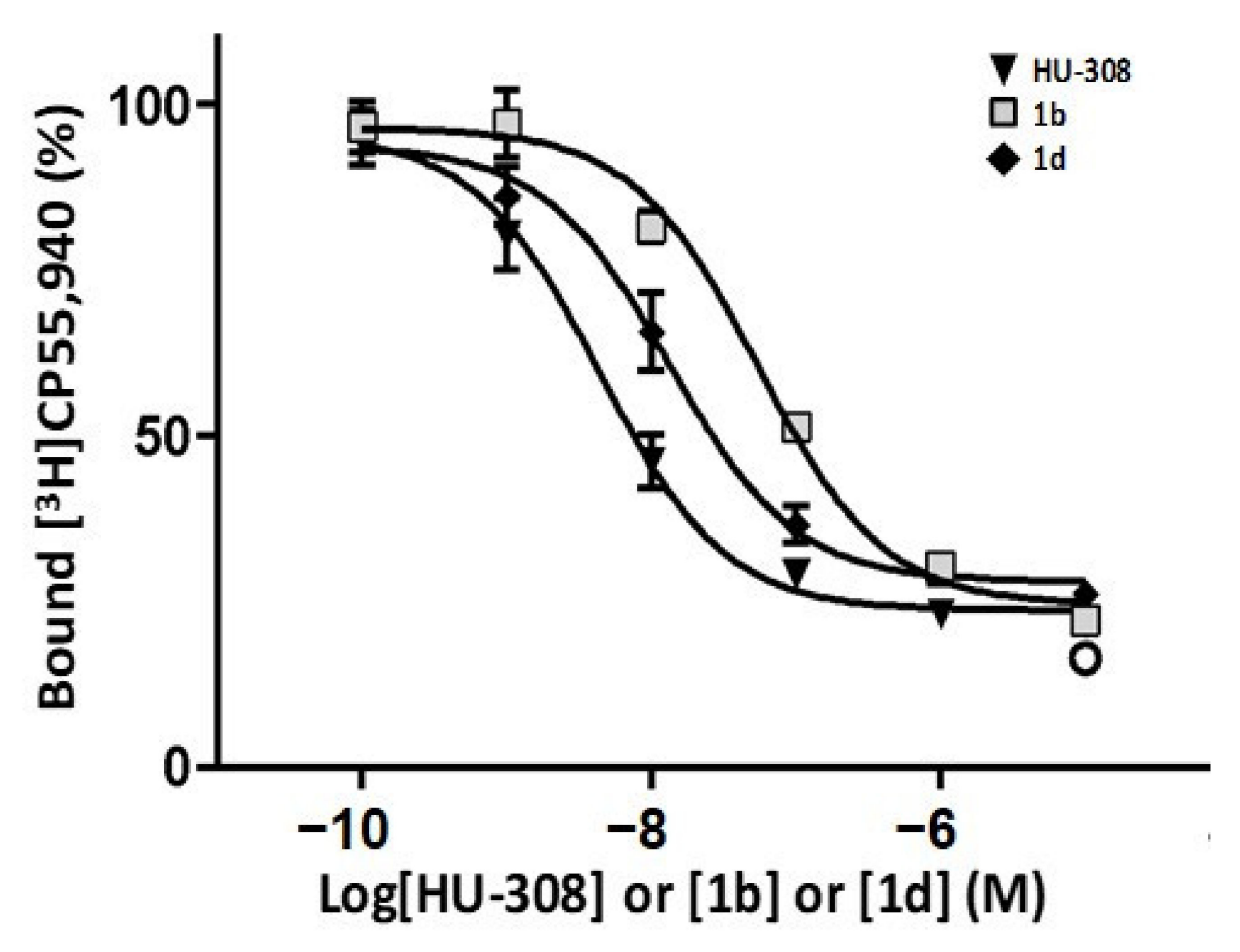
In vitro interaction of the agonists **HU-308**, **1b** and **1d** with hCB2R. Ki values for CB2R were derived from displacement of [^3^H]CP-55,940 binding in membranes of CHO-K1 cells expressing human CB2R. Each curve represents the mean of three experiments performed in triplicate.

As shown in Table 1, the fenchone derivatives showed high selectivity towards hCB2R over hCB1R. In most compounds, the (−) analogues prepared from the (−)-fenchone (**1b**, **1d**, **1f**, **2b**, **3b**, **3f**, **3h**, **4b**, **4d**, **5b** and **5d**) showed higher affinity towards hCB2R than their (+) counterparts prepared from (+)-fenchone (**1a**, **1c**, **1e**, **2a**, **3a**, **3e**, **3g**, **4a**, **4c**, **5a** and **5c**). We also observe that the affinity for the hCB2R can be optimized by varying the length of the side chain at C4′ for the fenchone-alkylresorcinol dimethyl ether derivatives (**1a–f**). Thus, the analogue with one methyl group at C4′ (**1f**) had the least affinity to hCB2R (K_i_ = 233 nM) compared to **1b** (with a pentyl group) and **1d** (with a dimethylheptyl group). Accordingly, **1a** (+ isomer) with a pentyl side chain displaced the binding of [^3^H]CP-55,940 to hCB2R with a K_i_ value of 47.7 nM and produced no measurable inhibition of [^3^H]CP-55,940 binding to hCB1R. On the other hand, **1b** (− isomer) inhibited [^3^H]CP-55,940 binding to hCB2R more strongly (K_i_ = 14.4 nM) with no detectable inhibition of [^3^H]CP-55,940 binding to hCB1R. Other potent compounds carry the dimethylheptyl substituent at C4′, which is typical for synthetic cannabinoids. **1c** (+ isomer) and **1d** (− isomer) with a dimethylheptyl side chain inhibited binding of [^3^H]CP-55,940 to hCB2R with a K_i_ value of 56.8 nM and 3.51 nM, respectively. The displacement of [^3^H]CP-55940 by **HU-308**, **1b** and **1d** from specific binding sites in membranes from cells expressing hCB2Rs is shown in Figure 4. The change in the position of the chain from the C4′ to C5′ (**2a** and **2b**) reduced the affinity towards hCB2R by an order of magnitude (K_i_ = 223 and 73.4 nM, respectively). The presence of only one methoxyl group in the aromatic part (**3a–h**) reduced the affinity to hCB2R dramatically (Table 1). Introducing a fluorine atom in the aromatic part of **1a–d** (**4a–d**) or demethylating it (**5a–d**) reduced the binding affinity of **1a–d** to the hCB2R by almost one order of magnitude (Table 1).

Analysis of the structure–activity relationships (SAR) of all of these analogs revealed several structural features for maintaining CB2R affinity and selectivity, including the stereochemistry of the compounds that should be the (−) derivatives, the presence of a branched lipophilic side chain at C4′, dimethoxy groups in the positions C2′ and C6′ and no substituents in the aromatic ring (Figure S4).

### 2.5. Functional Characterization

By using the [^35^S]GTPγS binding assay [24], we next evaluated the activity (agonism, antagonism and inverse agonism) properties of nine key compounds that showed the highest affinity for the hCB2R. Data are listed in Table 2 in which the CB2R agonist **HU-308** is included for comparison. Our testing results show that most compounds stimulate the GTPγS binding to CB2R, indicating that these compounds behaved as potent agonists at the hCB2R.

**Figure 5:**
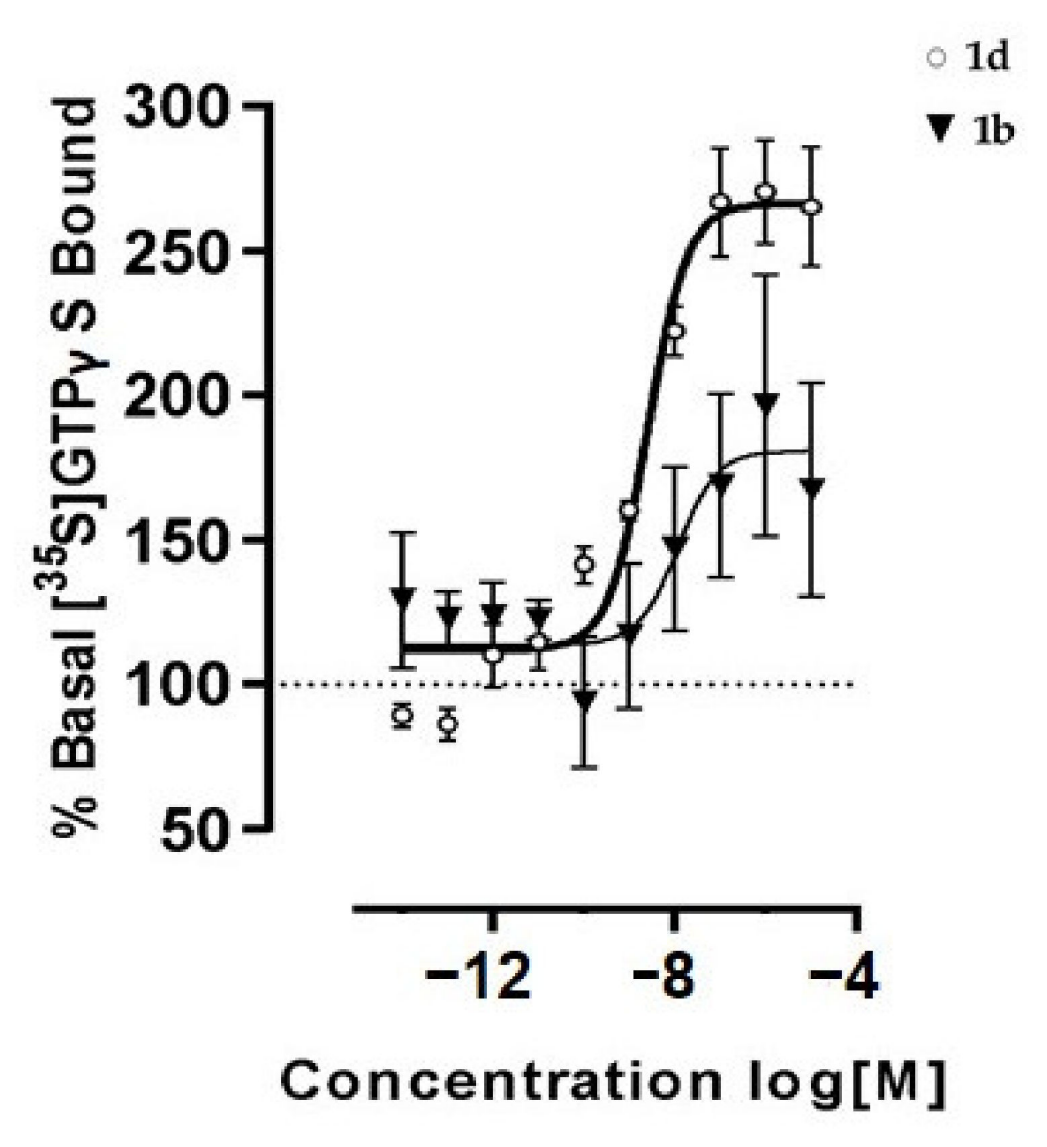
Mean log concentration–response curves of **1b** and **1d** for stimulation of [^35^S]GTPγS binding to hCB2R CHO cell membranes. Each symbol represents the mean percentage increase in [^35^S]GTPγS binding ± SE.

The (−) compounds with the dimethylheptyl side chains at C4′ (**1d**, **4d** and **5d**) were highly efficacious with **1d** being more potent (EC_50_ = 2.59 nM; E_(max)_ = 89.6%) than its monomethoxy (**5d)** (EC_50_ = 14.8 nM; E_(max)_ = 105%) and its fluorinated analogues (**4d)** (EC_50_ = 104 nM; E_(max)_ = 119%). The (−) compounds with the pentyl side chain at C4′ (**1b**, **4b** and **5b**) were less potent and less efficacious than their dimethylheptyl counterparts (Table 2) (Figure 5). Compound **2b** with a hexyl side chain in C5′ instead of C4′ weakly stimulated the [^35^S]GTPγS binding to hCB2R.

The most potent compounds in this assay, **1b**, **1d** and **5d**, appeared to be less potent and efficacious than **HU-308** at activating the hCB2R in the [^35^S]GTPγS binding assay (Table 2). However, the mean EC_50_ that it displayed in this assay was not significantly different from the corresponding EC_50_ values of **HU-308** as indicated by the overlap of the 95% confidence limits. Analysis of the SAR revealed that the structural features requirements for maintaining CB2R agonism are the same as those required for maintaining affinity and selectivity (Figure S4).

### 2.6. Effect on Inflammation and Hyperalgesia (Pain Sensation)

**Figure 6:**
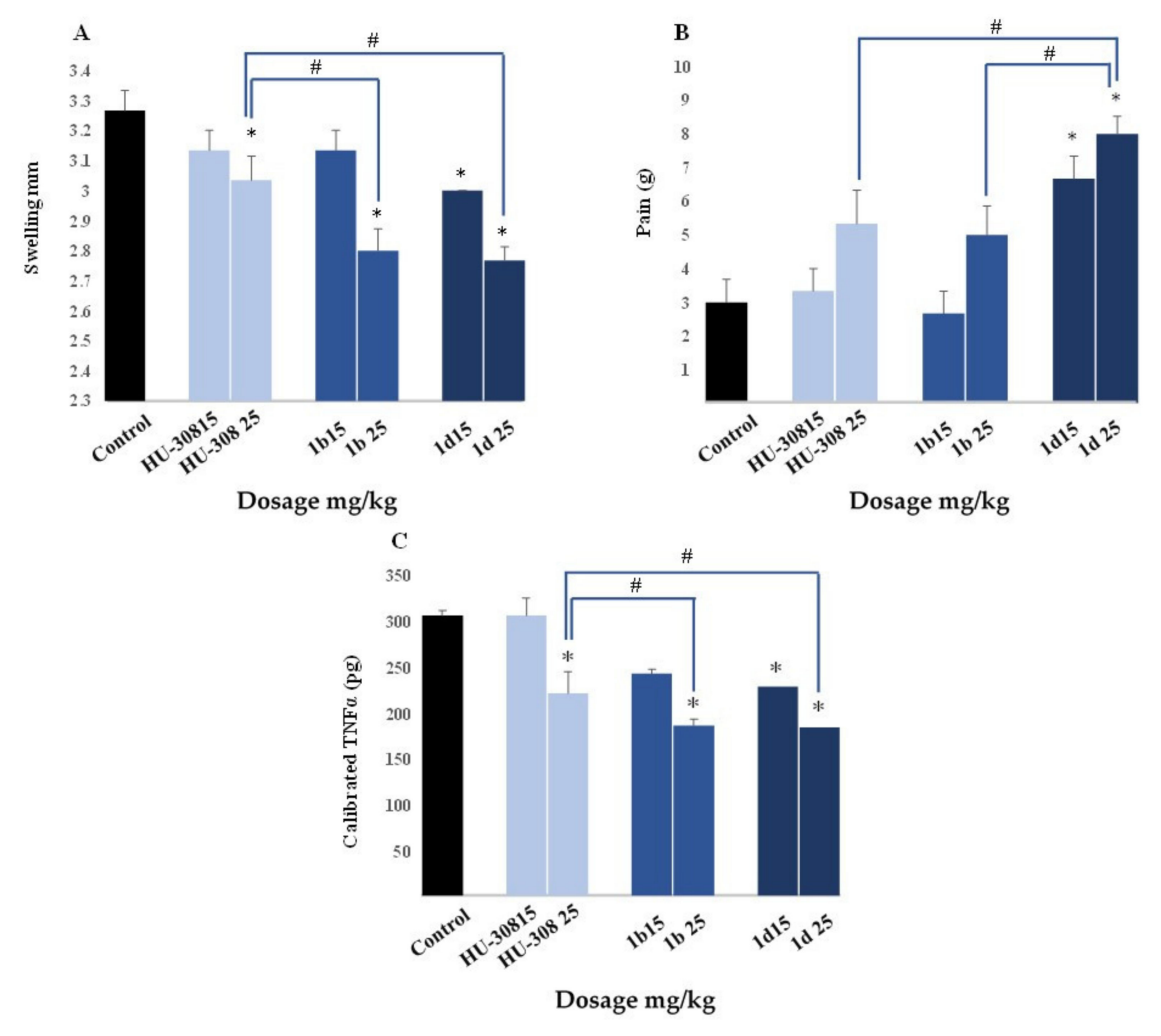
Anti-inflammatory and analgesic evaluation of **HU-308** (light blue) compared to **1b** (blue), **1d** (dark blue) and vehicle control (black). (**A**) Swelling after six hours, (**B**) pain sensation after six hours, and (**C**) TNF-α serum levels after 24 hours. Statistical comparison was done by 1-way ANOVA (*p*-value = 0.05). * *p* < 0.05 comparing to control group. # *p*-value < 0.05 in the indicated comparison.

Preclinical studies have shown that CB2R activation mitigates inflammatory reaction and swelling, indicating that CB2R agonists might be a beneficial target for treating inflammatory pain responses [25]. In this study, we used the zymosan-induced inflammation mouse model [26] to investigate the anti-inflammatory and anti-nociceptive activities of the fenchone derivatives, **1b** (the (−) enantiomer with a pentyl side chain at C4′) and **1d** (the (−) enantiomer with a dimethylheptyl side chain at C4′). The compounds were compared to **HU-308** and to a vehicle control group in three inflammation assays: paw swelling, pain sensation in the paw and circulating TNF-α (Figure 6).

The extent of hind paw swelling was determined 6 h following paw injection of 60 μg zymosan together with an ip administration of **HU-308**, **1b** or **1d** at two doses. The results revealed that both **1b** and **1d** have anti-inflammatory activity (Figure 6). **HU-308** was able to significantly reduce swelling at a dose of 25 mg/kg with 20% reduction in swelling (Figure 6A). However, both compounds, **1b** and **1d**, were able to significantly reduce swelling at the same concentration with 54% reduction in swelling and significantly exceed **HU-308**′s effect. More importantly, among the three compounds, only **1d** reduced swelling at 15 mg/kg with 30% reduction.

The anti-nociceptive effect was determined by the von Frey monofilament assay, and the higher the paw withdrawal threshold, the higher the anti-nociceptive effect of the drug. The two dosages, 15 and 25 mg/kg of **1d** showed strong anti-nociceptive effects after 6 h (*p* < 0.01) (Figure 6B). **1d** was significantly better than **1b** and **HU-308** at both doses (15 and 25 mg/kg) in reducing pain sensation. Moreover, the levels of TNF-α were reduced significantly for **HU-308** (25 mg/kg, 28% inhibition), **1b** (25 mg/kg, 39% inhibition) and **1d** (15 and 25 mg/kg with 26% and 40% inhibition, respectively) (Figure 6C). **1b** and **1d** were slightly but significantly better than **HU-308** in reducing TNF-α at 25 mg/kg and **1d** at 15 mg/kg was better than both compounds at the same concentration.

### 2.7. Molecular Modeling

**Figure 7:**
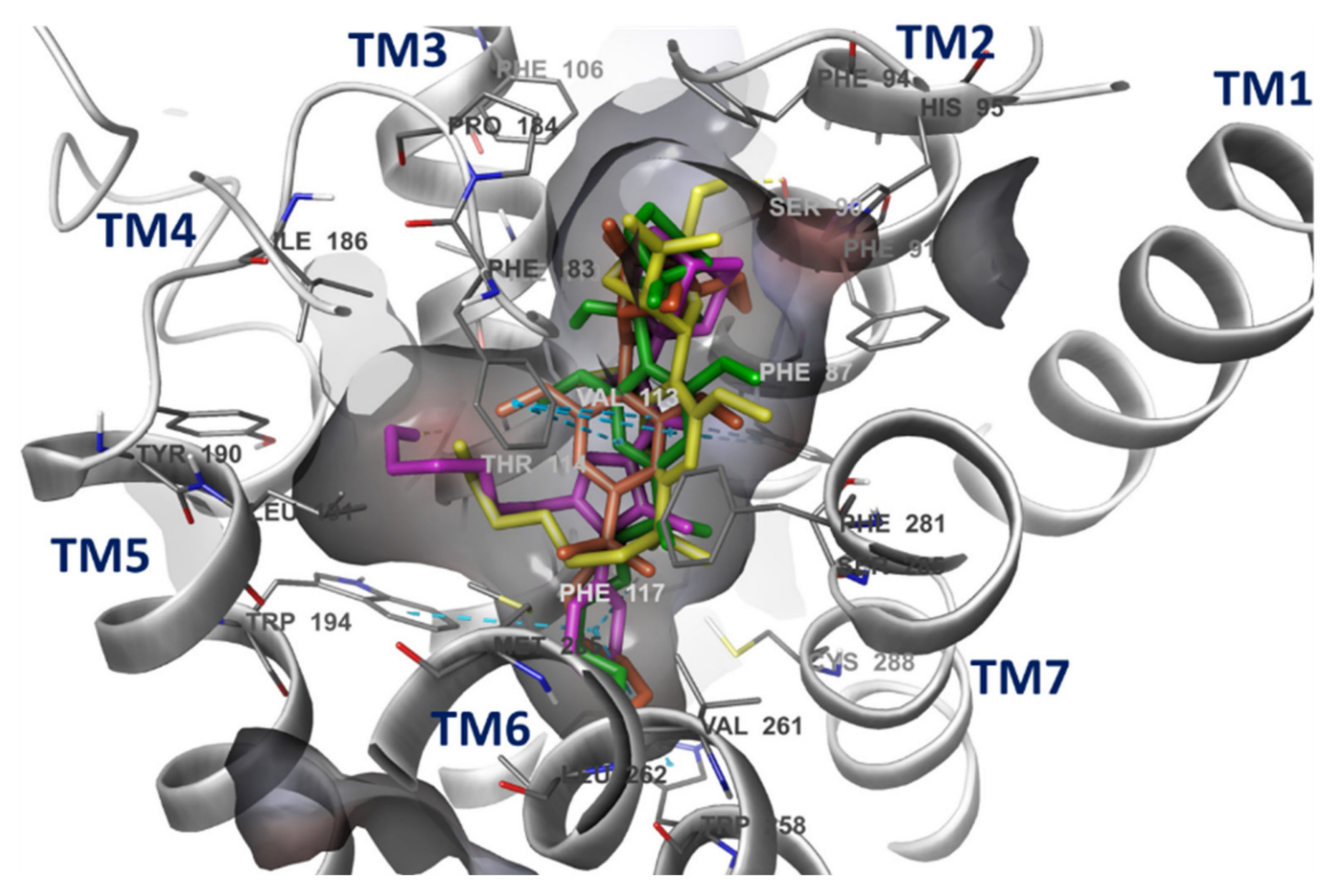
Orientation of docked ligands in the orthosteric site of CB2. The binding site of the CB2 cavity is represented by electrostatic potential surface. Ligands are shown in different colors; **5ZTY_ligand** (pink), **HU-308** (Yellow), **HU-433** (Orange) and **1d** (Green). TM means Transmembrane Helix. The residues of binding site and ligands are represented by thin and thick tubes, respectively. H-bonds and π–π interactions are represented by orange and cyan dotted lines, respectively.

We carried out molecular modeling studies to investigate the binding interactions of **1d** within the CB2 binding site and compare with the parent CB2 compounds, **HU-308** (+) and its (−) enantiomer, **HU-433**. For better understanding of the interactions and orientations of ligands in the orthosteric binding pocket, we superimposed all best docked poses of the compounds and colored them differently (Figure 7). The electrostatic potential surface shows the hydrophobic nature of the orthosteric site. In our previous studies [27], we reported that the enantiomers **HU-308** (+) and **HU-433** (−) adopted quite different poses in the binding site of CB2 receptor.

The **HU-308** has a constrained binding pose, while **HU-433** acquired the extending conformation. **1d** (-11.582 Kcal/mol) has the similar stereochemistry of **HU-433,** and therefore it adopted the extended conformation like **HU-433** (-11.848 Kcal/mol) in docking calculation. The interaction of CB2 with **1d** is mainly from the hydrophobic and aromatic residues of ECL2, TM2, TM3, TM4, TM5, TM6 and TM7. The bicyclic ring of **1d** establishes an extensive hydrophobic interaction network with the residues of extracellular side of the pocket, i.e., F91^2.61^, F94^2.64^, F95^2.65^, F106^3.25^ and I110^3.29^ while the 1,1-dimethylheptyl chain extends towards a deep pocket and forms hydrophobic interactions with the residues F117^3.36^, W194^5.53^, W258^6.48^, V261^6.51^, L262^6.52^ and F281^7.35^.

The affinity was further enhanced by the cooperative π–π interaction between the 2,6-dimethoxy phenyl ring of **1d** and F87^2.57^ and F183^ECL2^. The interaction of the ligands with CB2 are shown separately in Figures S5 and S6. The docking scores are shown in Table S3.

## 3. Conclusions

In the present study, we report the design and synthesis of a series of a novel class of CB2R selective ligands. These fenchone-resorcinol/phenol derivatives were synthesized and characterized by NMR, GCMS and LC-UV-MS (ESI). The X-ray data reinforced the structural elucidation and assignments of all NMR signals. Furthermore, the effects of different alkylresorcinols/phenols on the binding ability of these derivatives to the hCB1R and hCB2R were explored.

Structure–activity relationship studies (SAR) revealed several structural features for maintaining CB2R affinity and selectivity of these analogs. In particular, the (−)-fenchone-resorcinol compounds with branched lipophilic side chain at C4′, dimethoxy groups in the positions C2′ and C6′ and no substituents in the aromatic ring had high affinity and selectivity for the hCB2R. These structural features were also important for maintaining hCB2R agonism. Compound **1d** possessed high affinity (K_i_ = 3.51 nM) and selectivity for the hCB2R and was highly potent and efficacious hCB2R agonist (EC_50_ = 2.59 nM, E_(max)_ = 89.6%).

**1d** was potent in reducing the zymosan-induced paw swelling, pain responses and TNF-α levels in mice. Molecular-modeling analysis elucidated the binding interactions of **1d** within the CB2 binding site compared with the parent CB2 compounds, **HU-308** (+) and its (−) enantiomer, **HU-433**. Our data indicate that the design and the development of new terpenoid-derived cannabinoid-like drugs may play a significant role in human disease treatment.

## 4. Experimental Section

### 4.1. Chemistry

(+)-Fenchone (analytical standard, assay ≥99.5%, [α]20/D +62±1°, neat) and (1R)-(−)-Fenchone (≥98%, [α]24/D −50.5°, neat) were both purchased from Sigma-Aldrich. Other reagents and solvents were purchased from Biolab LTD (Jerusalem, Israel), J. T. Baker (Center Valley, PA, USA), Sigma-Aldrich (Rehovot, Israel), Acros (Yehud, Israel), Alfa Aesar (Lancashire, UK) and Merck (Darmstadt, Germany) and were used without further purification.

NMR spectra were recorded at 500 MHz (1D and 2D ^1^H and ^13^C NMR) for **1b, 1d** and **5a** and at 300 MHz (^1^H, ^13^C and ^19^F NMR) for the rest of the compounds using deuterated chloroform (CDCl_3_, δ = 7.26 ppm) with tetramethylsilane (TMS) as an internal standard. Thin-layer chromatography (TLC) was run on silica gel 60F_254_ plates (Merck). Column chromatography was performed on silica gel 60 Å (Merck). Compounds were located using a UV lamp at 254 nm. GCMS analyses were performed on an HP GCMS instrument (Model GCD PLUS) with an EI detector and a 30 m methyl silicone column. Optical rotations were measured on a polarimeter (Optical Activity) in a 2.00 dm cell at 25 °C.

#### 4.1.1. Synthesis of Fenchone-Resorcinol/Phenol

##### Methylation of Alkyl Phenols/Resorcinols

Methyl iodide (12 mmol) was added to a solution of alkyl phenol/resorcinol (1.51 mmol) and K_2_CO_3_ (12 mmol) in dry DMF (5 mL). After stirring at room temperature for 24 h, the mixture was diluted with water (40 mL) and extracted with ether. The organic layer was washed with water, dried and evaporated followed by purification by column chromatography on silica gel with ether/petroleum ether (2–4%).

##### Synthesis of 2-(2′,6′-Dimethoxy-4′-pentylphenyl)-1,3,3-trimethylbicyclo[2.2.1]heptan-2-ol (**1a** and **1b**)

To 0.5 g (2.4 mmol) 1,3-dimethoxy-5-pentylbenzene in 5 mL dry THF at room temperature was added n-BuLi (1.6 M in hexane, 3.3 mL, 5.28 mmol), and the resulting solution was refluxed under N_2_ for 2.5 h, cooled to r.t and the ketone (fenchone, 2.64 mmol, 0.401 g) in 1 mL THF was added. The fenchone used was the (+)-fenchone for 1a and (1R)-(−)-fenchone for 1b. The reaction mixture was refluxed for 3 h and then for 18 h at r.t. The reaction was worked up by the addition of saturated NH_4_Cl solution and extracted with ether. After washing (H_2_O) and drying (MgSO_4_), the solvent was evaporated to give the crude target compound, which was purified by chromatography, generally using ether/petroleum ether (3–5%).

Yield: 0.26 g (30%). White powder, mp= 76–78 °C. HPLC purity: 98.6%. ^1^H-NMR (500 MHz, CDCl_3_): 6.42 (s, 1H, H-3), 6.40 (s, 1H, H-5′), 3.79 (s, 3H, H-7′), 3.87 (s, 3H, H-8′), 2.57 (t, 2H, H-1″), 1.62 (m, 2H, H-2″), 1.37 (m, 4H, H-3″,H-4″), 0.93 (t, 3H, H-5″), 0.62 (s, 3H, H-7), 2.75 (dd, 1H, H-8), 1.12 (m, 5H, H-8, H-9, H-6), 1.17 (m, 4H, H-10, H-5), 2.41 (m, 1H, H-6), 1.71 (m, 2H, H-5, H-4). ^13^C-NMR (500 MHz, CDCl_3_): 159.16 (C-2′), 156.51 (C-6′), 142.23 (C-4′), 120.71 (C-1′), 105.59 (C-3′), 104.85 (C-5′), 55.57 (C-7′), 54.32 (C-8′), 35.91 (C-1″), 30.80 (C-2″), 31.64 (C-3″), 22.00 (C-4″), 14.07 (C-5″), 54.23 (C-1), 87.95 (C-2), 46.52 (C-3), 50.78 (C-4), 23.97 (C-5), 35.13 (C-6), 41.16 (C-8), 28.48 (C-7), 18.42 (C-10), 22.05 (C-9). LC-UV-MS (ESI): m/z calculated for C_23_H_36_O_3_ 360.27, found 343.09 (m-OH) for **1a** and 342.98 (m-OH) for **1b**. [α]_D_^25^ +112.7° for **1a** and –106.6° for **1b**.

##### Synthesis of 2-(2′,6′-Dimethoxy-4′-(2″-methyloctan-2″-yl)phenyl)-1,3,3-trimethylbicyclo[2.2.1]heptan-2-ol (**1c** and **1d**)

To a solution of the 1,3-dimethoxy-5-(2-methyloctan-2-yl)benzene (1 g, 3.8 mmol) in 32 mL of dry THF was added a 1.6 M solution of n-BuLi in hexane (8.8 mmol, 5.4 mL) at 0 °C with stirring under N_2_. After additional stirring for 1 h at 0 °C, a solution of the fenchone (6 mmol, 0.9 g) in 1 mL of dry THF was added all at once. The fenchone used was the (+)-fenchone for 1c and (1R)-(−)-fenchone for 1d. The reaction mixture was stirred for 0.5 h at 0 °C and then for 18 h at r.t. The reaction was worked up by the addition of saturated NH_4_Cl solution and extracted with ether. After washing (H_2_O) and drying (MgSO_4_), the solvent was evaporated to give the crude target compound, which was purified by chromatography with ether/petroleum ether (3–4%). Yield: 0.99 g (62.4%).

White powder, mp = 106–108 °C. HPLC purity: 98.9%. ^1^H-NMR (500 MHz, CDCl_3_): 6.50 (s, 1H, H-3′), 6.48 (s, 1H, H-5′), 3.86 (s, 3H, H-7′), 3.77 (s, 3H, H-8′), 1.55 (m, 2H, H-3″), 1.26–1.07 (m, 22 H, H-4″, H-5″, H-1″, H-9″, H-6″, H-7″, H-8, H-9, H-6, H-10), 0.84 (t, 3H, H-8″), 0.58 (s, 3H, H-7), 2.73 (dd, 1H, H-8), 1.35 (m, 1H, H-5), 2.38 (m, 1H, H-6), 1.68 (m, 2H, H-5, H-4). ^13^C-NMR (500 MHz, CDCl_3_): 158.90 (C-2′), 156.21 (C-6′), 149.14 (C-4′), 120.30 (C-1′), 103.33 (C-3′), 102.61 (C-5′), 55.52 (C-7′), 54.28 (C-8′), 37.74 (C-2″), 44.41 (C-3″), 22.62 (C-4″), 29.94 (C-5″), 31.70 (C-6″), 29.94 (C-7″), 14.07 (C-8″), 28.75 (C-1″), 28.75 (C-9″), 54.16 (C-1), 87.87 (C-2), 46.54 (C-3), 50.73 (C-4), 23.96 (C-5), 35.07 (C-6), 28.42 (C-7), 41.17 (C-8), 18.48 (C-9), 22.00 (C-10). LC-UV-MS (ESI): m/z calculated for C_27_H_44_O_3_ 416.33, found 399.12 (m-OH) for **1c** and 399.02 (m-OH) for **1d**. [α]_D_^25^ +96.88° for **1c** and −95.95° for **1d.**

##### Synthesis of 2-(2′,6′-Dimethoxy-4′-methylphenyl)-1,3,3-trimethylbicyclo[2.2.1]heptan-2-ol (**1e** and **1f**)

The same procedure as above but 1,3-dimethoxy-5-methylbenzene was used and no reflux was done. Yield: 0.16 g (22%). The fenchone used was the (+)-fenchone for 1e and (1R)-(−)-fenchone for 1f. White powder, mp = 90–92 °C. HPLC purity: 98.4%. ^1^H-NMR (300 MHz, CDCl_3_): 6.42 (s, 1H, H-3′), 6.40 (s, 1H, H-5′), 3.76 (s, 3H, H-7′), 3.85 (s, 3H, H-8′), 2.38 (t, 3H, H-1″), 0.60 (s, 3H, H-7), 2.72 (dd, 1H, H-8), 1.10 (m, 9H, H-8, H-9, H-10, H-6), 1.36 (m, 1H, H-5), 2.30 (m, 1H, H-6), 1.66 (m, 2H, H-5, H-4). LC-UV-MS (ESI): m/z calculated for C_19_H_28_O_3_ 304.20, found 287.05 (m-OH) for **1e** and **1f**. [α]_D_^25^ +91.5° for **1e** and −82.90° for **1f.**

##### Synthesis of 2-(3′-Hexyl-2′,6′-dimethoxyphenyl)-1,3,3-trimethylbicyclo[2.2.1]heptan-2-ol (**2a** and **2b**)

The same procedure as above but 1-hexyl-2,4-dimethoxybenzene was used and no reflux was done. Yield: 0.25 g (28%). The fenchone used was the (+)-fenchone for 2a and (1R)-(−)-fenchone for 2b. Yellowish powder, mp = 82–84 °C. HPLC purity: 98.6% for 2a and 96.4% for 2b. ^1^H-NMR (300 MHz, CDCl_3_): 7.06 (d, 1H, H-4′), 6.61 (d, 1H, H-5′), 3.81 (s, 3H, H-7′), 3.75 (s, 3H, H-8′), 2.57 (t, 2H, H-1″), 1.64 (m, 2H, H-2″), 1.51 (m, 2H, H-3″), 1.33 (m, 4H, H-4″, H-5″), 0.89 (t, 3H, H-6″), 0.73 (s, 3H, H-7), 2.82 (dd, 1H, H-8), 1.05 (m, 5H, H-8, H-9, H-6), 1.33 (m, 4H, H-10, H-5), 2.36 (m, 1H, H-6), 1.73 (m, 2H, H-5, H-4). LC-UV-MS (ESI): m/z calculated for C_24_H_38_O_3_ 374.28, found 357.08 (m-OH) for **2a** and **2b.** [α]_D_^25^ +84.82° for **2a** and −90.32° for **2b**

##### Synthesis of 2-(2′-Methoxy-5′-propylphenyl)-1,3,3-trimethylbicyclo[2.2.1]heptan-2-ol (**3a** and **3b**)

The same procedure as for **1a** and **1b** but 1-methoxy-4-propylbenzene was used. Yield: 0.38 g (53%). The fenchone used was the (+)-fenchone for 3a and (1R)-(−)-fenchone for 3b. White solid, mp = 78–80 °C. HPLC purity: 98.8%. ^1^H-NMR (300 MHz, CDCl_3_): 7.33 (s, 1H, H-6′), 6.80 (d, 1 H, H-3′), 7.00 (d, 1 H, H-4′), 3.86 (s, 3H, H-7′), 2.54 (m, 2H, H-1″), 1.57 (m, 2H, H-2″), 0.93 (t, 3H, H-3″), 0.46 (s, 3H, H-7), 2.54 (m, 1H, H-8), 1.12 (m, 5H, H-8, H-9, H-6), 1.30 (m, 4H, H-10, H-5), 2.23 (m, 1H, H-6), 1.73 (m, 2H, H-5, H-4). LC-UV-MS (ESI): m/z calculated for C_20_H_30_O_2_ 302.22, found 285.07 (m-OH) for **3a** and **3b**. [α]_D_^25^ +63.44° for **3a** and −62.96° for **3b**.

##### Synthesis of 2-(2′-Methoxy-5′-pentylphenyl)-1,3,3-trimethylbicyclo[2.2.1]heptan-2-ol (3c and **3d**)

The same procedure as for **1a** and **1b** but 1-methoxy-4-pentylbenzene was used. Yield: 0.20 g (25%). The fenchone used was the (+)-fenchone for 3c and (1R)-(−)-fenchone for 3d. White solid, mp = 42–44 °C. HPLC purity: 93.8%. ^1^H-NMR (300 MHz, CDCl_3_): 7.31 (s, 1H, H-6′), 6.77 (d, 1H, H-3′), 6.94 (d, 1H, H-4′), 3.81 (s, 3H, H-7′), 2.52 (m, 2H, H-1″), 1.57 (m, 2H, H-2″), 1.39 (m, 4H, H-3″,H-4″), 0.89 (t, 3H, H-5″), 0.45 (s, 3H, H-7), 2.54 (m, 1H, H-8), 1.17 (m, 5H, H-8, H-9, H-6), 1.29 (m, 4H, H-10, H-5), 2.24 (m, 1H, H-6), 1.72 (m, 2H, H-5, H-4). LC-UV-MS (ESI): m/z calculated for C_22_H_34_O_2_ 330.26, found 313.09 (m-OH) for **3c** and **3d**. [α]_D_^25^ +99.29° for **3c** and −96.99° for **3d**.

##### Synthesis of 2-(2′-Methoxy-5′-(tert-pentyl)phenyl)-1,3,3-trimethylbicyclo[2.2.1] heptan-2-ol (**3e** and **3f**)

The same procedure as for **1a** and **1b** but 1-methoxy-(4-*tert*-pentyl)benzene was used. Yield: 0.35 g (44%). The fenchone used was the (+)-fenchone for 3e and (1R)-(−)-fenchone for 3f. White solid, mp = 68–70 °C. HPLC purity: 96.2%. ^1^H-NMR (300 MHz, CDCl_3_): 7.48 (s, 1H, H-6′), 6.81 (d, 1H, H-3′), 7.09 (d, 1H, H-4′), 3.86 (s, 3H, H-7′), 1.62 (m, 2H, H-3″), 0.66 (t, 3H, H-4″), 0.43 (s, 3H, H-7), 2.48 (m, 1H, H-8), 1.16 (m, 5H, H-8, H-9, H-6), 1.36 (m, 4H, H-10, H-5), 2.22 (m, 1H, H-6), 1.73 (m, 2H, H-5, H-4). LC-UV-MS (ESI): m/z calculated for C_22_H_34_O_2_ 330.26, found 313.09 (m-OH) for **3e** and 313.10 (m-OH) for **3f**. [α]_D_^25^ +81.07° for **3e** and −82.68° for **3f**.

##### Synthesis of 2-(2′-Methoxy-5′-octylphenyl)-1,3,3-trimethylbicyclo[2.2.1]heptan-2-ol (**3g** and **3h**)

The same procedure as for **1a** and **1b** but 1-methoxy-4-(2-methylheptan-2-yl)benzene was used. Yield: 0.18 g (20%). The fenchone used was the (+)-fenchone for 3g and (1R)-(−)-fenchone for 3h. White solid: 120–122 °C. HPLC purity: 95.5% for 3g and 92.4% for 3h. ^1^H-NMR (300 MHz, CDCl_3_): 7.52 (s, 1H, H-6′), 6.78 (d, 1H, H-3′), 7.13 (d, 1H, H-4′), 3.86 (s, 3H, H-7′), 2.64 (m, 2H, H-1″), 1.70 (m, 2H, H-2″), 1.43 (m, 10H, H-3″,H-4″,H-5″,H-6″,H-7″), 0.73 (t, 3H, H-8″), 0.44 (s, 3H, H-7), 2.45 (m, 1H, H-8), 1.12 (m, 5H, H-8, H-9, H-6), 1.34 (m, 4H, H-10, H-5), 2.23 (m, 1H, H-6), 1.70 (m, 2H, H-5, H-4). LC-UV-MS (ESI): m/z calculated for C_25_H_40_O_2_ 372.30, found 355.12 (m-OH) for **3g** and **3h**. [α]_D_^25^ 88.52° for **3g** and 81.82° for **3h**.

#### 4.1.2. Fluorination with Selectflour^®^

We dissolved 0.48 mmol of Selectflour^®^ in 2.6 mL acetonitrile. The solution was cooled to 0–5 °C and fenchone-dimethoxyalkylresorcinol (0.48 mmol) in 2.6 mL acetonitrile was added to it. The reaction was stirred at this temperature for 1.5 h, and then the reaction was left stirring overnight at room temperature. Ether was added to the reaction mixture, and then the mixture was washed with brine. Removal of the solvent afforded the desired products, which were purified by chromatography with ether/petroleum ether (0–2%) [19].

##### 2-(3′-Fluoro-2′,6′-dimethoxy-4′-pentylphenyl)-1,3,3-trimethylbicyclo[2.2.1]heptan-2-ol (**4a** and **4b**)

The fenchone used was the (+)-fenchone for 4a and (1R)-(−)-fenchone for 4b. Yield: 0.031 g (17%). White solid, mp = 70–72 °C. HPLC purity: 97.2% for 4a and 97.3% for 4b. ^1^H-NMR (300 MHz, CDCl_3_): 6.37 (s, 1H, H-5′), 3.92 (s, 3H, H-8′), 3.97 (s, 3H, H-7′), 2.55 (t, 2H, H-1″), 1.66 (m, 2H, H-2″), 1.32 (m, 4H, H-3″, H-4″), 0.88 (t, 3H, H-5″), 0.61 (s, 3H, H-7), 2.71 (dd, 1H, H-8), 1.12 (m, 5H, H-8, H-9, H-6), 1.16 (m, 4H, H-10, H-5), 2.48 (m, 1H, H-6), 1.58 (m, 2H, H-5, H-4). ^19^F-NMR (300 MHz, CDCl_3_): −140. LC-UV-MS (ESI): m/z calculated for C_23_H_35_FO_3_ 378.26, found 361.09 (m-OH) for **4a** and 360.91 (m-OH) for **4b**. [α]_D_^25^ 110.01° for **4a** and 103.13° for **4b**.

##### 2-(3′-fluoro-2′,6′-dimethoxy-4′-(2″-methyloctan-2″-yl)phenyl)-1,3,3-trimethylbicyclo[2.2.1]heptan-2-ol (**4c** and **4d**)

The fenchone used was the (+)-fenchone for 4c and (1R)-(−)-fenchone for 4d. Yield: 0.035 g (22%). White solid: 100–102 °C. HPLC purity: 90%. ^1^H-NMR (300 MHz, CDCl_3_): 6.41 (s, 1H, H-5′), 3.96 (s, 3H, H-7′), 3.90 (s, 3H, H-8′), 1.57 (m, 2H, H-3″), 1.26 (m, 10 H, H-4″, H-5″, H-1″, H-9″), 1.21 (m, 4H, H-6″,H-7″), 0.84 (t, 3H, H-8″), 0.61 (s, 3H, H-7), 2.75 (dd, 1H, H-8), 1.08 (m, 5H, H-8, H-9, H-6), 1.15 (m, 3H, H-10), 1.35 (m, 1H, H-5), 2.40 (m, 1H, H-6), 1.72 (m, 2H, H-5, H-4). ^19^F-NMR (CDCl_3_): −134.6. LC-UV-MS (ESI): m/z calculated for C_27_H_43_FO_3_ 434.32, found 417.13 (m-OH) for **4c** and 417.12 (m-OH) for **4d**. [α]_D_^25^ 69.98° for **4c** and 58.30° for **4d**.

#### 4.1.3. Demethylation with Sodium Ethanethiolate/DMF

We added 4–8 mL (2–4 mmol) of a 0.5 M solution of NaSEt in DMF to the aromatic methoxy compound (1 mmol), and the resulting solution was heated in an oil bath at 115–120 °C under N_2_. The completion of the reaction in each case was determined by TLC. The cooled reaction mixture was then acidified with 10% aqueous HCl and extracted with EtOAc (3 × 10 mL). The combined organic extracts were washed with 10% aqueous NaOH (3 × 3 mL) and H_2_O (3 mL) and dried (MgSO_4_). Removal of the solvent afforded the desired products, which were purified by chromatography with ether/petroleum ether (6–8%) [22].

##### 2-(2′-hydroxy-6′-methoxy-4′-pentylphenyl)-1,3,3-trimethylbicyclo[2.2.1]heptan-2-ol (**5a** and **5b**)

The fenchone used was the (+)-fenchone for 5a and (1R)-(−)-fenchone for 5b. Yield: 0.045 g (13%). White powder: 76–78 °C. HPLC purity: 98.7% for 5a and 100% for 5b. ^1^H-NMR (500 MHz, CDCl_3_): 6.38 (s, 1H, H-3′), 6.40 (d, 1H, H-5′), 3.77 (s, 3H, H-7′), 2.51 (t, 2H, H-1″), 1.63 (m, 2H, H-2″), 1.36 (m, 4H, H-3″, H-4″), 0.92 (t, 3H, H-5″), 0.71 (s, 3H, H-7), 2.82 (dd, 1H, H-8), 1.18 (m, 5H, H-8, H-9, H-6), 1.20 (s, 4H, H-10, H-5), 2.41 (m, 1H, H-6), 2.05 (m, 1H, H-6), 1.74 (m, 2H, H-5, H-4). ^13^C-NMR (500 MHz, CDCl_3_): 159.42 (C-6′), 156.40 (C-2′), 143.58 (C-4′), 115.00 (C-1′), 110.96 (C-5′), 102.85 (C-3′), 54.32 (7′), 35.54 (C-1″), 30.52 (C-2″), 31.69 (C-3″), 22.55 (C-4″), 14.06 (C-5″), 55.05 (C-1), 90.86 (C-2), 47.55 (C-3), 50.03 (C-4), 23.30 (C-5), 35.23 (C-6), 41.33 (C-8), 27.90 (C-7), 17.61 (C-10), 22.00 (C-9). LC-UV-MS (ESI): m/z calculated for C_22_H_34_O_3_ 346.25, found 328.99 (m-OH) for **5a** and 328.94 (m-OH) for **5b**. [α]_D_^25^ 56.25° for **5a** and 48.01° for **5b**

##### 2-(2′-Hydroxy-6′-methoxy-4′-(2″-methyloctan-2″-yl)phenyl)-1,3,3-trimethylbicyclo-[2.2.1]heptan-2-ol (**5c** and **5d**)

The fenchone used was the (+)-fenchone for 5c and (1R)-(−)-fenchone for 5d. Yield: 0.048 g (12%). White powder: 98–100 °C. HPLC purity: 90%. ^1^H-NMR (300 MHz, CDCl_3_): 6.50 (s, 1H, H-5′), 6.48 (s, 1H, H-3′), 3.86 (s, 3H, H-7′), 1.55 (m, 2H, H-3″), 1.26 (m, 10 H, H-4″, H-5″, H-1″, H-9″), 1.20 (m, 4H, H-6″, H-7″), 0.84 (t, 3H, H-8″), 0.58 (s, 3H, H-7), 2.73 (dd, 1H, H-8), 1.07 (m, 5H, H-8, H-9, H-6), 1.15 (m, 3H, H-10), 1.35 (m, 1H, H-5), 2.38 (m, 1H, H-6), 1.68 (m, 2H, H-5, H-4). ^13^C-NMR (300 MHz, CDCl_3_): 158.90 (C-6″), 156.21 (C-2″), 149.14 (C-4″), 120.30 (C-5″), 118.20 (C-1″), 103.33 (C-3″), 55.52 (C-7″), 37.74 (C-2″), 44.41 (C-3″), 22.62 (C-4″), 29.94 (C-5″), 31.70 (C-6″), 29.94 (C-7″), 14.07 (C-8″), 28.75 (C-1″), 28.75 (C-9″), 54.16 (C-1), 87.87 (C-2), 46.54 (C-3), 50.73 (C-4), 23.96 (C-5), 35.07 (C-6), 28.42 (C-7), 41.17 (C-8), 18.48 (C-9), 22.00 (C-10). LC-UV-MS (ESI): m/z calculated for C_26_H_42_O_3_ 402.31, found 385.13 (m-OH) for **5c** and 384.97 (m-OH) for **5d**. [α]_D_^25^ +96.88° for **1c** and −95.95° for **1d**.

### 4.2. X-ray Crystallography

A single crystal of the compound was attached to a 400/50 MicroMeshes™ with NVH Oil [28] and transferred to a Bruker SMART APEX CCD X-ray diffractometer equipped with a graphite-monochromator. The system was controlled by a pentium-based PC running the SMART software package [29]. MoKα radiation (λ = 0.71073 Å) was used for data collection at room temperature, and CrysAlisPro [30] was used for data processing using Olex2 [31]. The SHELXT [32] structure solution program using Intrinsic Phasing was used for structure solving, and the SHELXL [33] refinement package using Least Squares minimization was used for structure refinement.

### 4.3. Binding Assays

Binding to the CB1R was assessed in a competition displacement assays using [^3^H]CP-55,940 as the radioligand [24]. Membranes from cells expressing hCB1R and hCB2R were purchased from Charles River, (Cat#A317 and A318, respectively; OH, USA). Solutions of test compounds ranging from 0.1 nM to 10 mM were prepared in DMSO. The desired amount of membrane preparation was diluted with ice-cold assay buffer (50 mM Tris-HCl, 2.5 mM EDTA, 5 mM MgCl_2_, 0.1% BSA, pH 7.4) and was vortexed. We distributed 100 µL of compound into each tube, followed by addition of 800 µL of diluted membranes (1 µg/tube) and kept on ice until the addition of [^3^H]CP-55,940. [^3^H]CP-55,940 was diluted with cold (unlabeled) assay buffer and 100 µL was added into each tube. The assays were incubated for 90 min at 30 °C and then immediately filtered on WHATMAN GF/B Filter Paper (Fired) using a Brandel M-24R Harvester followed by six washes with ice cold wash buffer (50 mM Tris-HCl, 2.5 mM EDTA, 5 mM MgCl_2_, 0.1% BSA, pH 7.4). Radioactivity was detected by adding the Filter Paper directly to the ULTIMA GOLD scintillation cocktail (PerkinElmer, Waltham, MA, USA), incubation at 20 °C for 60 min and then counted using a Tri-Carb 4910TR liquid scintillation counter (PerkinElmer, Waltham, MA, USA).

### 4.4. [^35^S]GTPγS Binding Assay

The method used for measuring agonist-stimulated binding of [^35^S]GTPγS was based on a described protocol [24]. Membranes (5 μg protein) were incubated in GTPγS assay buffer (50 mM Tris HCl (pH 7.4), 0.2 mM EGTA, 9 mM MgCl_2_, 150 mM NaCl, 1 mg/mL BSA) containing 100 μM GDP, 0.05 nM [^35^S]GTPγS and test compounds at various concentrations. Vacuum filtration was used to separate bound ligand from free ligand. We used 10 μM GTPS to determine nonspecific binding. Basal binding was assayed in the absence of the ligand and in the presence of GDP.

### 4.5. Biological Evaluation

#### 4.5.1. Animals

Female Sabra mice, 6 to 8-weeks old were kept in the SPF unit of Hadassah Medical School. They were maintained at a 20–21 °C and a 12 h light/dark cycle with a standard pellet diet and water ad libitum. The experimental protocols were approved by the Animal Care Ethical Committee of the Hebrew University-Hadassah Medical School, Jerusalem, Israel. The data presented in the Figures are representatives of two separate experiments.

#### 4.5.2. Paw Inflammation and Treatment with **HU-308/1b/1d**

We injected 40 μL of 1.5% (*w/v*) zymosan A in 0.9% saline into the right hind paw of the mice to induce inflammation. HU-308/1b/1d in 0.1 mL ethanol:Cremophore:saline (1:1:18) (vehicle) was injected intraperitoneally (i.p.) immediately after zymosan injection. Control mice were injected with vehicle. Paw swelling and pain perception were measured after 2, 6 and 24 h [26].

#### 4.5.3. Measurement of Oedema Formation

The paw swelling (thickness) was measured by calibrated calipers (0.01 mm), 2, 6 and 24 h following injections of zymosan alone or HU-308/1b/1d [26].

#### 4.5.4. Pain Assay

The paw withdrawal von Frey test was used to evaluate hyperalgesia at 2, 6 and 24 h following injections of zymosan and/or the test compounds [26]. In the von Frey nociceptive filament assay, von Frey calibrated monofilament hairs of logarithmically incremental stiffness (1.4–60 g corresponding to 4.17 to 5.88 log of force) was used to test the mouse sensitivity to a mechanical stimulus on the swollen paw. The measurements were done in a quiet room. The animals were held for 10 s prior to paw pain measurements. The investigator applied the filament to the central area of the hind paw with gradual increasing size. The middle of the hind paw was poked to provoke a flexion reflex followed by a clear flinch response after paw withdrawal. Each filament was applied for 3–4 s to induce the end-point reflex. The force filament of 1.4 g was used for the first testing. In the case of no withdrawal response, the next higher stimulus was tried. The mechanical threshold force (in grams (g)) was defined as the lowest force imposed by two von Frey monofilaments of various sizes, required to produce a paw retraction. The untreated left hind paw served as a control.

#### 4.5.5. Measurement of TNFα

After 24 h of zymosan injection, blood was collected, and the sera were assayed for TNFα. A mouse TNFα ELISA kit was used according to the manufacturer’s instructions (R&D Systems, Minneapolis, MN, USA).

### 4.6. Molecular Modelling Studies

The three-dimensional structure of human CB2 (PDB ID: 5ZTY) was downloaded from the protein databank. The missing residues between 222 and 235 were modelled, and mutations were reverted to wildtype residues. The protonation states of all acidic and basic residues were assigned at physiological pH 7.2. The retrained minimization considering 0.30 Å root mean square deviation (RMSD) of all atoms was performed using optimized potentials for a liquid simulation extended (OPLS3e) force field.

All docking calculations were performed using two different docking protocols, Schrodinger suit 2020.3 [34] and Autodock 4.5.7 [35]. The orthosteric ligand binding site was defined by generating 20 Å grid around the co-crystallized small molecule (AM10257) in Glide, whereas 60 × 60 × 60 grid points with a 0.375 Å spacing around the centroid of AM10257 were generated in Autodock.

The compounds were prepared at pH 7.0 ± 2.0 using the LigPrep module. The docking calculations were performed using the default protocol of GLIDE module. The 10 conformations of each compound were generated using Standard precision (SP) docking. The 10 poses of each conformation were generated using Extra precision (XP) docking. The best pose was selected based on the lowest energy and interaction with the active site residues. Lamarckian Genetic Algorithm was used in Autodock to identify the binding poses of each compound.

The receptor was kept rigid, whereas the ligand was allowed torsional flexibility. The default parameters were set but with 2.5 × 10^7^ energy evaluations. The 50 poses of each compound were generated using the Lamarckian Genetic Algorithm. The resulting poses were clustered into groups of 2.0 Å root-mean-square deviation (rmsd). The best scoring pose from the group with a higher number of conformers was chosen as the final pose. Both software demonstrated similar lowest energy poses of the ligands.

### 4.7. Statistical Analyses

Statistical analysis was performed with GraphPad Prism software. Statistical analysis details are listed under each figure. The results are presented as the value ± SE (standard error). In rare cases where all the measurements give the same values, no SE bar is presented, as no error can be measured. * *p* < 0.05 comparing to control group. # *p*-value < 0.05 in the indicated comparison.

## Figures and Tables

**Table 1:** Affinities of fenchone derivatives for hCB1R and hCB2R.

Compound	K_i_ (nM) hCB1R	95% Confidence Interval (nM)	K_i_ (nM) hCB2R	95% Confidence Interval (nM)
**1a**	NB	-	47.7	(21.2–107)
**1b**	>10 µM	-	14.4	(9.27–22.5)
**1c**	NB	-	56.8	(39.4–81.9)
**1d**	>10 µM	-	3.51	(2.07–5.96)
**1e**	NB	-	>10 µM	-
**1f**	NB	-	233	(171–317)
**2a**	NB	-	223	(126–397)
**2b**	>10 µM	-	73.4	(48.0–112)
**3a**	NB	-	1012	(376–2723)
**3b**	NB	-	610	(44.6–8343)
**3c**	NB	-	444	(246–800)
**3d**	NB	-	834	(414–1681)
**3e**	NB	-	2874	(150–54,940)
**3f**	NB	-	1449	(135–15,540)
**3g**	NB	-	1651	(65.4–41,720)
**3h**	NB	-	494	(171–1427)
**4a**	NB	-	155	(13.5–1787)
**4b**	>10 µM	-	28.3	(8.31–96.5)
**4c**	NB	-	438	(246–781)
**4d**	>10 µM	-	56.6	(30.9–104)
**5a**	NB	-	4978	(841–29470)
**5b**	>10 µM	-	36.5	(17.0–78.4)
**5c**	NB	-	107	(55.7–207)
**5d**	>10 µM	-	24.5	(16.2–37.3)
**HU-308**	NB	-	1.16	(0.71–1.90)

NB = No binding detected at concentrations up to 10 µM. >10 µM = Displacement of radioactive ligand detected at high concentrations competing ligand.

**Table 2:** Functional potencies (EC_50_) of key fenchone derivatives and **HU-308** for the hCB2R (±SE confidence limits).

Compound	EC_50_(nM) ^1^	95% Confidence Interval (nM)	*E(max)*% ^2^
**1b**	11.6 (agonist)	(2.25–70.9)	65.1
**1c**	121 (agonist)	(13.6–676)	87.1
**1d**	2.59 (agonist)	(1.01–6.75)	89.6
**2b**	1943 (agonist)	(98.0–…)	46.2
**4b**	305 (agonist)	(113–807)	54.2
**4d**	104 (agonist)	(56.2–186)	119
**5b**	82.8 (agonist)	(31.9–193)	69.5
**5d**	14.8 (agonist)	(7.80–29.2)	105
**HU-308**	1.01 (agonist)	(0.0330–8.94)	100

^1^ Functional potencies at hCB2R were determined by measuring the increase in [^35^S]GTPγS binding to the receptor. EC_50_ values were calculated using nonlinear regression analysis. Data are the average of two independent experiments run in triplicate. ^2^ *E(max)* values (maximal effects) is presented as the percentage of **HU-308** response at 1 µM.

## Data Availability

The data presented in this study are available on request from the corresponding author.

## Supplementary Materials

The following supporting information are available online: Figure S1: The HSQC of compound **1d**; Figure S2: Crystal structures of **1b** and **4b**; Figure S3: Crystal structures of **1d** and **5d**; Table S1: Crystal data and structure refinement for **1b**, **4b**, **1d**, and **5d**; Table S2: Hydrogen bonds for **1b**, **4b**, **1d**, and **5d** (Å, u); Figure S4: Structural Requirements for CB2 affinity and selectivity; Figure S5: Binding site of the CB2 cavity is represented by electrostatic potential surface. The residues of binding site and ligands are represented by thin and thick tubes respectively. H-bonds and π-π interactions are represented by orange and cyan dotted lines respectively. A. **5ZTY_Ligand** B. **HU-308** C. **HU-433** D. **1d**; Figure S6: Two dimensional representation of protein-ligand interactions. A. **5ZTY_Ligand** B. **HU-308** C. **HU-433** D. **1d**; Table S3: Docking Glide XP score with energy decomposition; LC-UV-MS (ESI); NMR data for **1b**; NMR data for **1d**; NMR data for **5a**.

## Sample Availability

Samples of the compounds are not available from the authors.
