# Supplementary material for: Fenchone Derivatives as a Novel Class of CB2 Selective Ligands: Design, Synthesis, X-ray Structure and Therapeutic Potential
Source: Molecules. 2022 Feb 18;27(4):1382. doi: 10.3390/molecules27041382 (PMC8878464; doi:10.3390/molecules27041382)
Supplement: Supplementary file 1 [file molecules-27-01382-s001.zip › molecules-1567005-supplementary.pdf]

Supplementary Material

1. Figure S1: The HSQC of compound **1d**
2. Figure S2: Crystal structures of **1b** and **4b**
3. Figure S3: Crystal structures of **1d** and **5d**
4. Table S1: Crystal data and structure refinement for **1b**, **4b, 1d**, and **5d**
5. Table S2: Hydrogen bonds for **1b**, **4b, 1d**, and **5d** (Å, u)
6. Figure S4: Structural Requirements for CB2 affinity and selectivity
7. Figure S5: Binding site of the CB2 cavity is represented by electrostatic potential surface. The residues of binding site and ligands are represented by thin and thick tubes respectively. H-bonds and π-π interactions are represented by orange and cyan dotted lines respectively. A. **5ZTY_Ligand** B. **HU-308** C. **HU-433** D. **1d**.
8. Figure S6: Two dimensional representation of protein-ligand interactions. A. **5ZTY_Ligand** B. **HU-308** C. **HU-433** D. **1d**.
9. Table S3: Docking Glide XP score with energy decomposition
10. LC-UV-MS (ESI)
11. NMR data for **1b**
12. NMR data for **1d**
13. NMR data for **5a**

1d

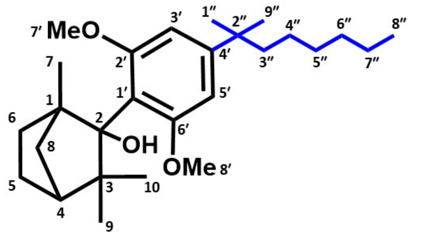

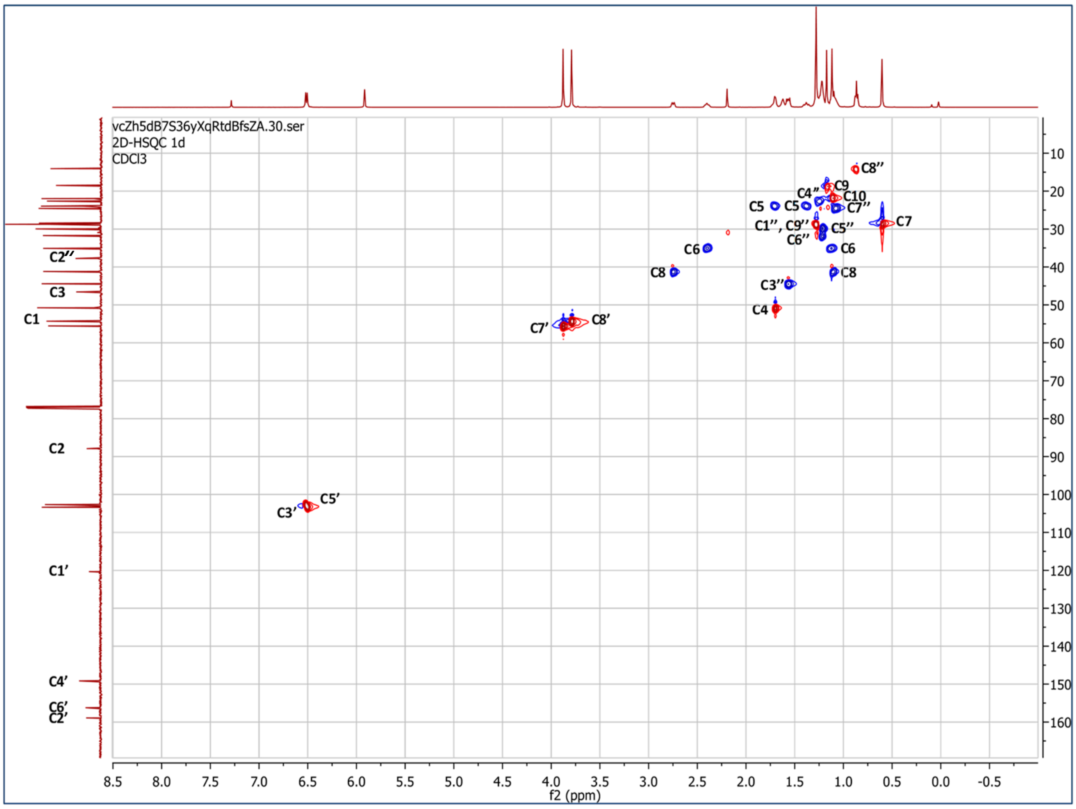

Figure S1: The HSQC of compound **1d**

The cross peaks in the 2D-HSQC confirm which 13C and 1H are coupled via a one-bond scalar coupling. Thus, the quaternary carbon peaks with no directly attached protons in the molecule do not give any cross-peaks. The carbon peaks at about 158.90, 156.21, 149.14, 120.30, 54.16, 87.87, 46.54 and 37.74 ppm in the f1 dimension display quaternary carbon atoms without detectable cross-peaks. These peaks were assigned to the aromatic carbons C-2′, C-6′, C-4′, C-1′, the cyclic carbons C-1, C-2, C-3 and the aliphatic carbon C-2″ respectively. The assignments of the two aromatic protons of H-3′ and H-5′ (6.50 and 6.48 ppm) were confirmed by their cross-peaks to the aromatic carbons C-3′ and C-5′ (103.33 and 102.61 ppm). Similarly, the two nonequivalent protons of H-7′ and H-8′ (3.86 and 3.77 ppm) were confirmed by cross-peaks to the carbon atoms C-7′ and C-8′ (55.52 and 54.28 ppm). The assignments of the two nonequivalent protons of H-8 (2.73 and 1.26-1.07 ppm) were confirmed by their cross-peaks to the same carbon resonance (41.17 ppm). Similarly, the two nonequivalent protons of H-6 (2.38 and 1.26-1.07 ppm) were confirmed by cross-peaks to the same carbon resonance (35.07 ppm). Also, the two nonequivalent protons of H-5 (1.68 and 1.35 ppm) were confirmed by cross-peaks to the same carbon resonance (23.96 ppm). All the other protonated carbons were assigned in a straightforward manner.

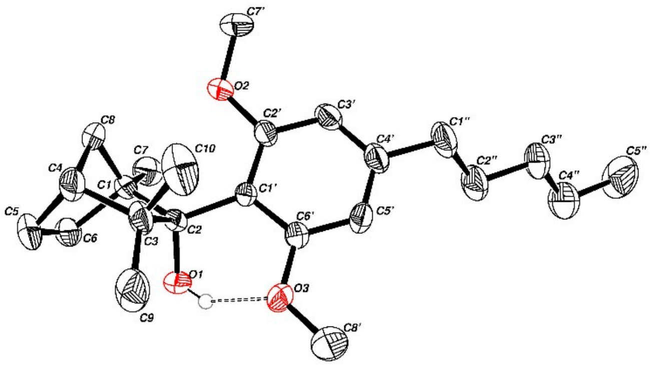

1b

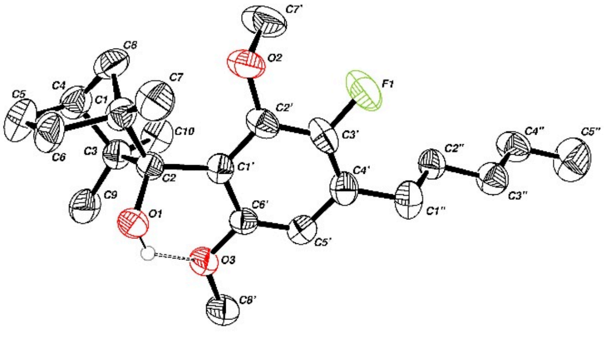

4b

Figure S2: Crystal structures of **1b** and **4b**

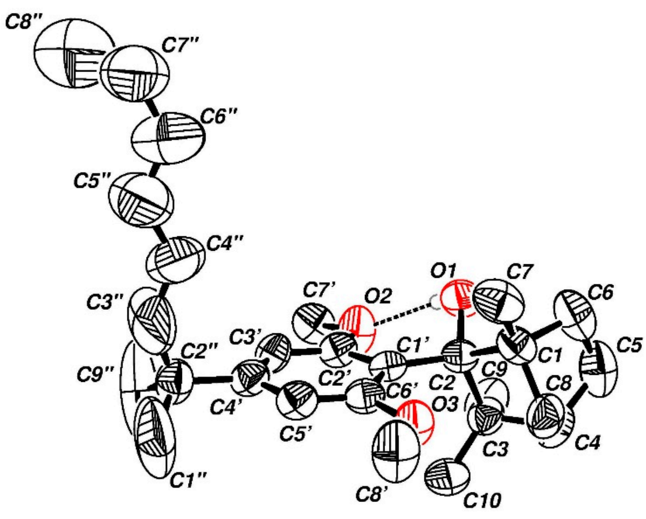

1d

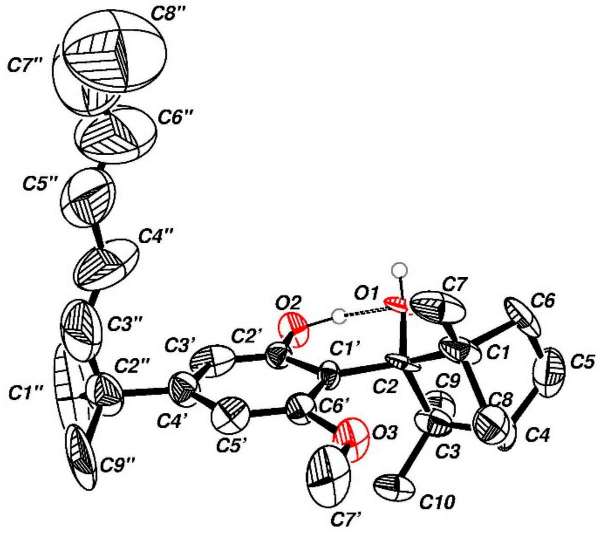

5d

Figure S3: Crystal structures of **1d** and **5d**

The bond length of C(3')-H(3') in **1b** was 0.97(2) A° while that of C(3')-F(1) in **4b** was 1.365(4) Å (Figure 2S). The bond lengths were within normal ranges (1.09 Å for C-H bond [1] and 1.35 Å for C-F bond [2]). The C-F bond length was slightly longer than C-H. The angles of C(2')-C(3')-H(3') and C(2')-C(3')-F(1) were 119.3(1)º and 116.7(4)º, respectively. In both compounds, **1b** and **4b**, the molecules were interlinked by intermolecular hydrogen bonds. Regarding **1d** and **5d** (Figure 3S), the replacement of a methoxyl group with a hydroxyl group on the C(2') position of the aromatic ring increased the O(1)-C(2)-C(1) angle by 4º [from 103.3(3) to 107.2(4)] and decreased the O(1)-C(2)-C(3) dihedral angle by 5.5º [from 112.7(3) to 107.2(5)]. In **1d**, the hydrogen bond was O(1)-H(1)···O(3). However, when this compound was demethylated (**5d**), the hydrogen bond changed to O(2)-H(2) ···(O1) and O(1)-H(1) made a hydrogen bond to O(2) of neighboring molecule.

1. Handbook of Chemistry & Physics (65th ed.). CRC Press.
2. O'Hagan, D. Understanding organofluorine chemistry. An introduction to the C–F bond. *Chem. Soc. Rev*. **2008**, 37, 308–319.

Table S1: Crystal data and structure refinement for **1b**, **4b, 1d** and **5d**

	**1b**	**4b**	**1d**	**5d**
Empirical formula	$C_{23}H_{36}O_3$	$C_{23}H_{35}F\ O_3$	$C_{27}\ H_{44}\ O_3$	$C_{26}\ H_{42}\ O_3$
Formula weight	360.52	378.51	416.62	402.60
Temperature [K]	293(1)	295(1)	293(1)	293(1)
Wavelength	0.71073	0.71073	0.71073	0.71073
Crystal system	Monoclinic	Orthorhombic	Orthorhombic	Orthorhombic
Space group	P2(1)/n	P2(1)2(1)2(1)	P2(1)2(1)2(1)	P2(1)2(1)2(1)
Unit cell dimensions				
a [Å]	9.6585(5)	7.9475(5)	7.9490(4)	7.8307(5)
b [Å]	8.9094(4)	12.9307(8)	12.8962(6)	12.4716(7)
c [Å]	24.8603(1)	21.046(1)	24.934(1)	25.775(2)
α [°]	90.00	90.00	90.00	90.00
β [°]	98.589(5)	90.00	90.00	90.00
γ [°]	90.00	90.00	90.00	90.00
Volume [Å^3]	2115.3(2)	2162.8(2)	2556.1(2)	2517.2(3)
Z	4	4	4	4
$Density_{calcd}$ [mg/m^3]	1.132	1.162	1.083	1.062
Absorption coefficient [mm^{-1}]	0.073	0.081	0.068	0.067
F(000)	792	824	920	888
Crystal size [mm^3]	0.44 x 0.40 x 0.16	0.54 x 0.11 x 0.09	0.65 x 0.50 x 0.32	0.54 x 0.30 x 0.09
2Θ range for data collection/°	4.338 to 53.996	3.87 to 53.996	4.544 to 54	3.628 to 49.984
Index ranges	-12<=h<=12	-10<=h<=10	-10<=h<=10	-9<=h<=9
	-11<=k<=11	-16<=k<=16	-16<=k<=16	-14<=k<=14
	-31<=l<=31	-26<=l<=26	-31<=l<=31	-30<=l<=30
Reflections collected	21747	22791	27024	22451
Independent reflections	4626 [$R_{(int)}$ = 0.0444]	4721 [R(int) = 0.0790]	5576 [R(int) = 0.0737]	4435 [R(int) = 0.0840]
Data / restraints / parameters	4626/0/253	4721/0/254	5576/0/283	4435/0/277
Goodness-of-fit on F^2	1.103	1.150	1.072	1.115
Final R indices [I>2σ(I)]; R_1, wR_2	0.0663, 0.1628	0.0679, 0.1204	0.0804, 0.2040	0.0906, 0.1781
R_1, wR_2 (all data)	0.0817, 0.1722	0.1064, 0.1373	0.0971, 0.2160	0.1359, 0.1969
Largest diff. peak and hole [e.Å^{-3}]	0.25/-0.22	0.22/-0.21	0.29/-0.30	0.21/-0.17

Table S2: Hydrogen bonds for **1b**, **4b, 1d**, and **5d** (Å, u)

**Cpd**	D-H...A	d(D-H)	d(H...A)	d(D...A)	**<(DHA)**
**1b**	O(1)-H(1)...O(3)	0.85(3)	1.78(3)	2.531(2)	147(3)
**4b**	O(1)-H(1)...O(3)	0.89(5)	1.81(5)	2.564(4)	141(5)
**1d**	O(1)-H(1)...O(3)	0.87(6)	1.75(6)	2.518(5)	145(5)
**5d**	O(2)-H(2)...O(1)	0.95(7)	1.66(7)	2.483(6)	142(6)
	O(1)-H(1)...O(2)#	0.92(7)	1.93(7)	2.795(6)	156(6)

Symmetry transformations used to generate equivalent atoms: #1 1/2+X,1/2-Y,1-Z

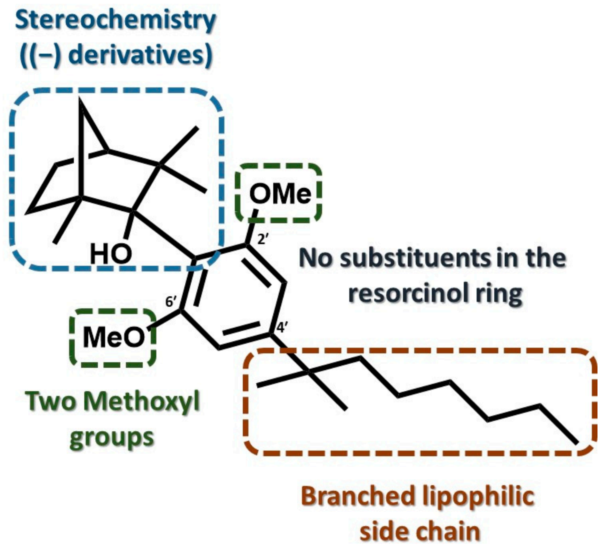

Figure S4: Structural Requirements for CB2 affinity and selectivity

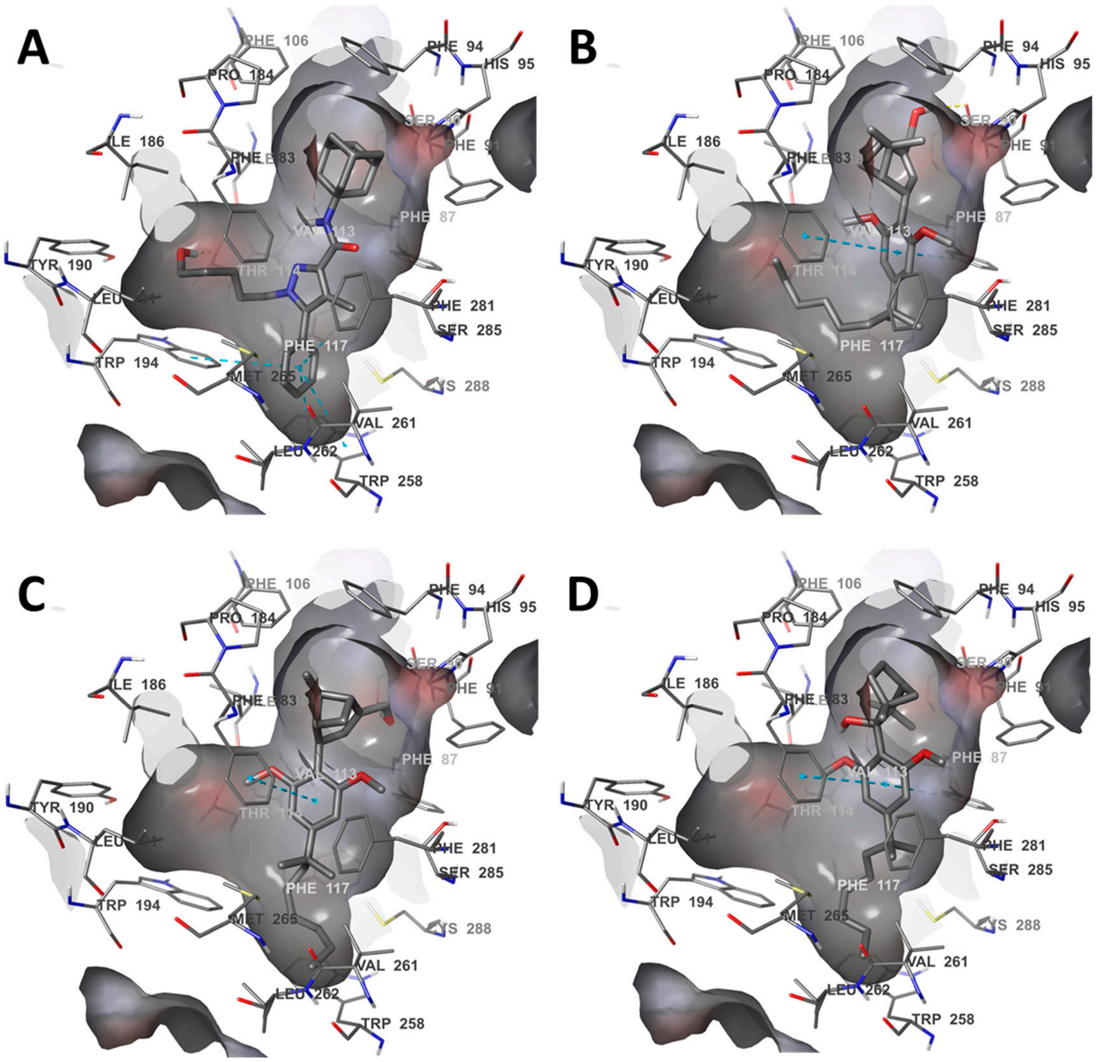

Figure S5: Binding site of the CB2 cavity is represented by electrostatic potential surface. The residues of binding site and ligands are represented by thin and thick tubes respectively. H-bonds and π-π interactions are represented by orange and cyan dotted lines respectively. A. **5ZTY_Ligand** B. **HU-308** C. **HU-433** D. **1d.**

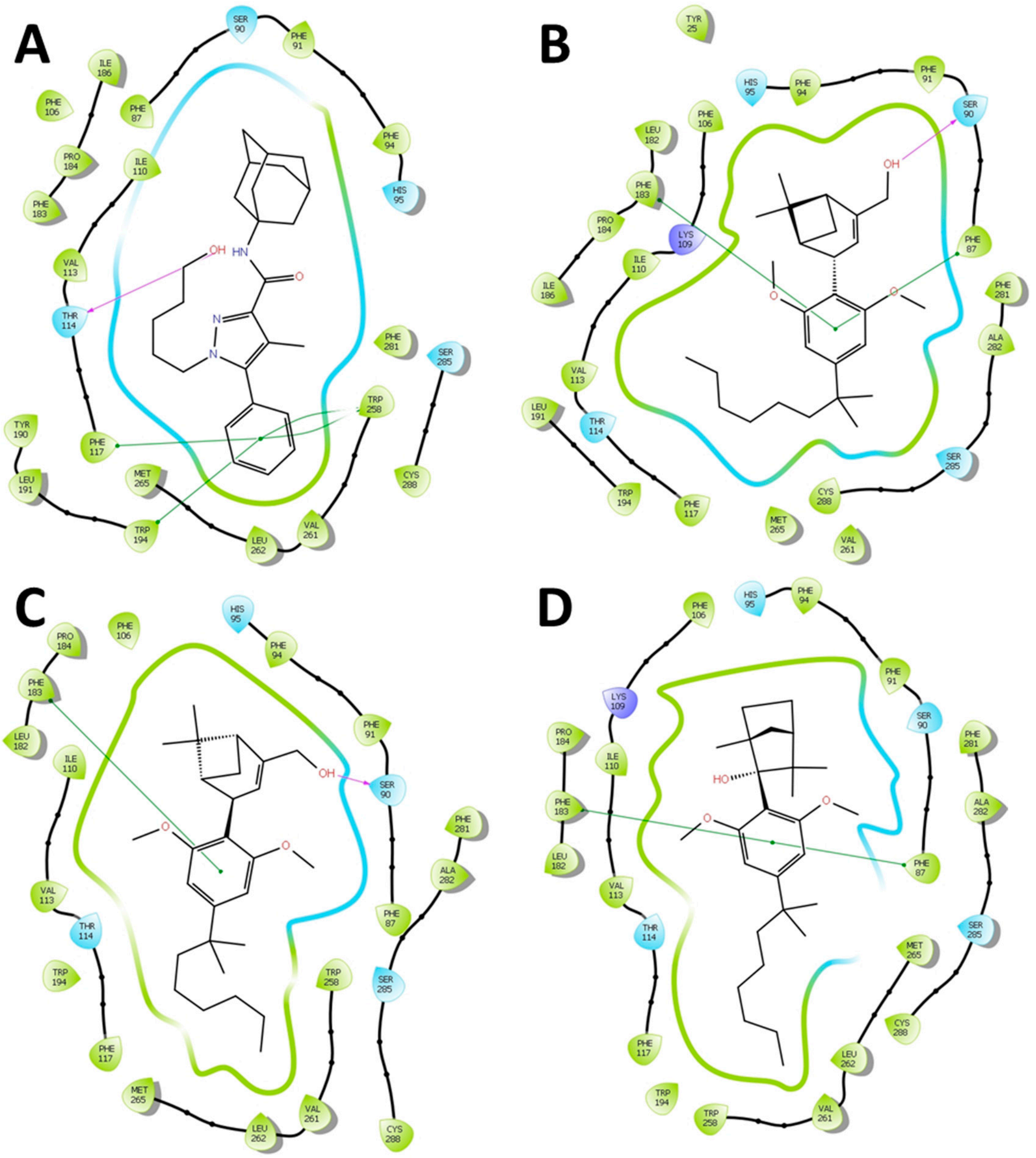

Figure S6: Two dimensional representation of protein-ligand interactions. A. **5ZTY_Ligand** B**. HU-308** C. **HU-433** D. **1d**.

Table S3: Docking Glide XP score with energy decomposition

**Compound**	**Ki (nM) hCB2R**	XP GScore (Kcal/mol)	XP HBond	XP PhobEn	XP LowMW	XP RotPenal	XP LipophilicEvdW	XP Electro
**1a**	47.7	-10.578	-0.480	-2.700	-0.298	0.253	-7.288	-0.065
**1b**	14.45	-10.352	-0.480	-2.700	-0.298	0.253	-7.091	-0.035
**1c**	56.81	-11.301	-0.480	-2.700	-0.111	0.293	-8.301	-0.001
**1d**	3.509	-11.582	-0.480	-2.700	-0.111	0.293	-8.534	-0.049
**1e**	>10 μM	-9.573	-0.480	-2.700	-0.485	0.085	-5.918	-0.075
**1f**	233.1	-9.562	-0.480	-2.700	-0.485	0.085	-5.890	-0.092
**2a**	223.5	-10.747	-0.480	-2.700	-0.251	0.295	-7.578	-0.032
**2b**	73.38	-10.730	-0.480	-2.700	-0.251	0.295	-7.502	-0.092
**3a**	1012	-9.714	-0.480	-2.700	-0.492	0.173	-6.169	-0.046
**3b**	610.2	-9.962	-0.480	-2.700	-0.492	0.173	-6.368	-0.095
**3c**	444.1	-9.930	-0.480	-2.700	-0.398	0.295	-6.551	-0.096
**3d**	834.2	-10.088	-0.480	-2.700	-0.398	0.295	-6.707	-0.098
**3e**	2874	-9.812	0.000	-2.700	-0.398	0.148	-6.800	-0.062
**3f**	1449	-10.162	-0.480	-2.700	-0.398	0.148	-6.637	-0.094
**3g**	1651	-10.265	-0.480	-2.700	-0.258	0.298	-7.102	-0.023
**3h**	494.3	-10.893	-0.480	-2.700	-0.258	0.298	-7.710	-0.043
**4a**	155.4	-10.684	-0.480	-2.700	-0.238	0.232	-7.157	-0.051
**4b**	28.33	-10.560	-0.480	-2.700	-0.238	0.232	-7.000	-0.074
**4c**	438.4	-11.606	-0.480	-2.700	-0.051	0.271	-8.468	-0.039
**4d**	56.56	-11.418	-0.480	-2.700	-0.051	0.271	-8.150	-0.075
**5a**	4978	-10.404	-0.480	-2.700	-0.298	0.316	-7.140	-0.103
**5b**	36.53	-11.027	-0.960	-2.700	-0.298	0.316	-7.343	-0.042
**5c**	107.4	-11.455	-0.960	-2.700	-0.158	0.311	-7.917	-0.032
**5d**	24.57	-11.199	-0.960	-2.700	-0.158	0.311	-7.609	-0.084
**HU-308**	1.161	-11.754	-0.638	-2.700	-0.118	0.344	-8.483	-0.160
**HU-433**		-11.848	-0.700	-2.700	-0.118	0.344	-8.507	-0.168

LC-UV-MS (ESI)

2-(2′,6′-dimethoxy-4′-pentylphenyl)-1,3,3-trimethylbicyclo[2.2.1]heptan-2-ol (1a & 1b) $C_{23}H_{36}O_3$

m/z calculated for 360.27, found 343.09 (m-OH) for **1a** and 342.98 (m-OH) for **1b**.

2-(2′,6′-dimethoxy-4′-(2″-methyloctan-2″-yl)phenyl)-1,3,3-trimethylbicyclo-[2.2.-1]heptan-2-ol (1c & 1d) $C_{27}H_{44}O_3$

m/z calculated 416.33, found 399.12 (m-OH) for **1c** and 399.02 (m-OH) for **1d**.

2-(2′,6′-dimethoxy-4′-methylphenyl)-1,3,3-trimethylbicyclo[2.2.1]heptan-2-ol (1e & 1f) $C_{19}H_{28}O_3$

m/z calculated 304.20, found 287.05 (m-OH) for **1e** and **1f**.

2-(3′-hexyl-2′,6′-dimethoxyphenyl)-1,3,3-trimethylbicyclo[2.2.1]heptan-2-ol (2a & 2b) $C_{24}H_{38}O_3$

m/z calculated 374.28, found 357.08 (m-OH) for **2a** and **2b**.

2-(2′-methoxy-5′-propylphenyl)-1,3,3-trimethylbicyclo[2.2.1]heptan-2-ol (3a & 3b) $C_{20}H_{30}O_2$

m/z calculated 302.22, found 285.07 (m-OH) for **3a** and **3b**.

2-(2′-methoxy-5′-pentylphenyl)-1,3,3-trimethylbicyclo[2.2.1]heptan-2-ol (3c & 3d) $C_{22}H_{34}O_2$

m/z calculated 330.26, found 313.09 (m-OH) for **3c** and **3d**.

2-(2′-methoxy-5′-(tert-pentyl)phenyl)-1,3,3-trimethylbicyclo[2.2.1]heptan-2-ol (3e & 3f) $C_{22}H_{34}O_2$

m/z calculated 330.26, found 313.09 (m-OH) for **3e** and 313.10 (m-OH) for **3f**.

2-(2′-methoxy-5′-octylphenyl)-1,3,3-trimethylbicyclo[2.2.1]heptan-2-ol (3g & 3h) $C_{25}H_{40}O_2$

m/z calculated 372.30, found 355.12 (m-OH) for **3g** and **3h**.

2-(3′-fluoro-2′,6′-dimethoxy-4′-pentylphenyl)-1,3,3-trimethylbicyclo[2.2.1]heptan-2-ol (4a & 4b) $C_{23}H_{35}FO_3$

m/z calculated 378.26, found 361.09 (m-OH) for **4a** and 360.91 (m-OH) for **4b**.

2-(3′-fluoro-2′,6′-dimethoxy-4′-(2″-methyloctan-2″-yl)phenyl)-1,3,3-trimethyl-bicyclo[2.2.1]heptan-2-ol (4c & 4d) $C_{27}H_{43}FO_3$

m/z calculated 434.32, found 417.13 (m-OH) for **4c** and 417.12 (m-OH) for **4d**.

2-(2'-hydroxy-6′-methoxy-4′-pentylphenyl)-1,3,3-trimethylbicyclo[2.2.1]heptan-2-ol (5a & 5b) $C_{22}H_{34}O_3$

m/z calculated 346.25, found 328.99 (m-OH) for **5a** and 328.94 (m-OH) for **5b**.

2-(2′-hydroxy-6′-methoxy-4′-(2″-methyloctan-2″-yl)phenyl)-1,3,3-trimethyl-bicyclo[2.2.1]heptan-2-ol (5c & 5d) $C_{26}H_{42}O_3$

m/z calculated 402.31, found 385.13 (m-OH) for **5c** and 384.97 (m-OH) for **5d**.

Compound 1a, UV-247 nm

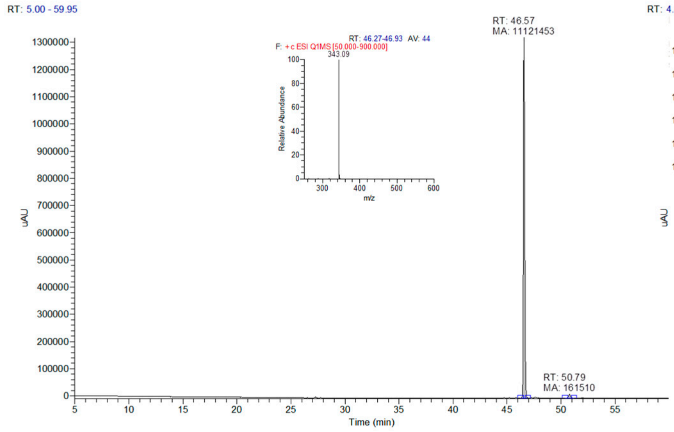

Compound 1b, UV -245nm

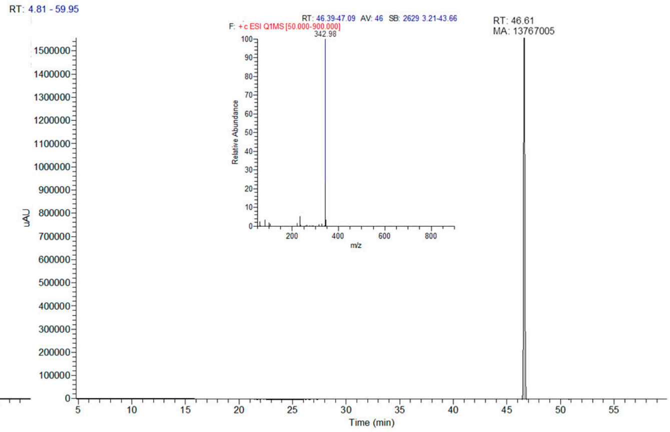

Compound 1c, UV-245 nm

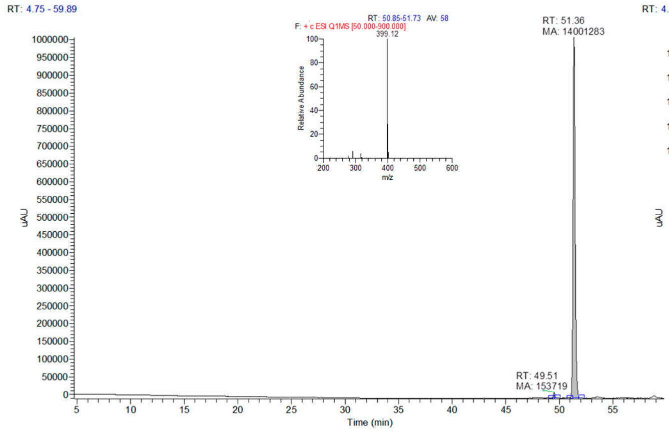

Compound 1d, UV-242 nm

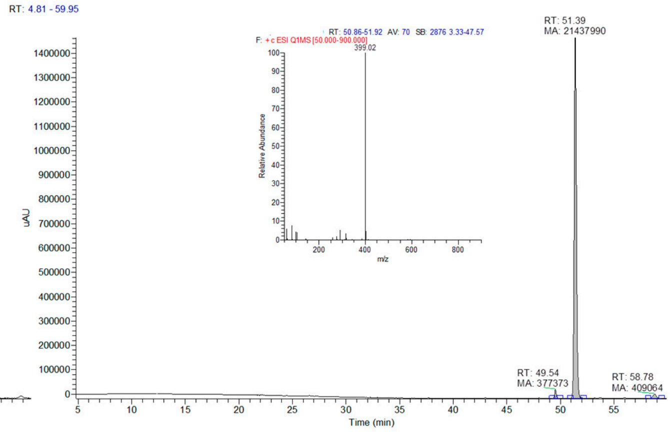

Compound 1e, UV-245 nm

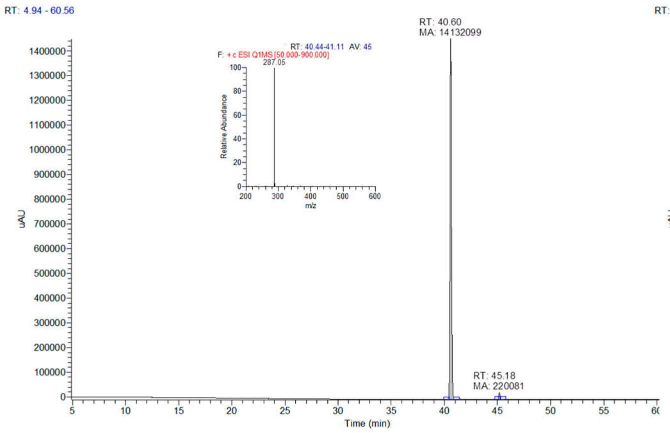

Compound 1f, UV-245 nm

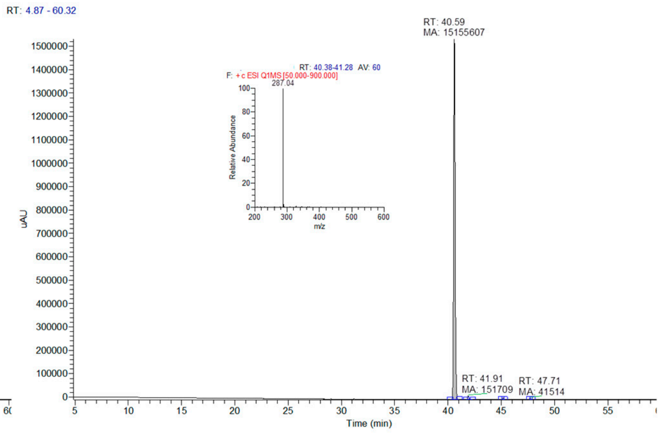

Compound 2a, UV-240 nm

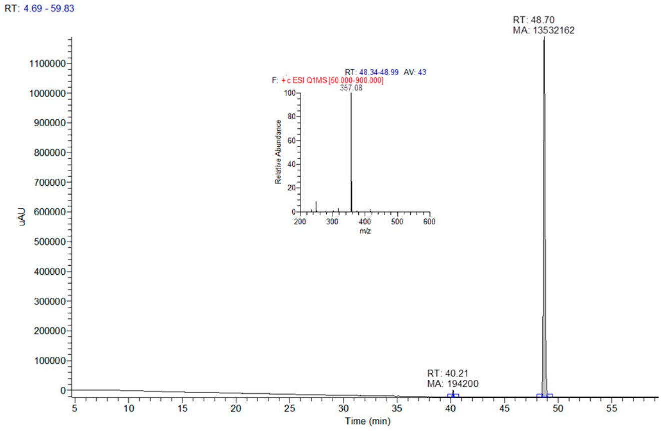

Compound 2b, UV-254 nm

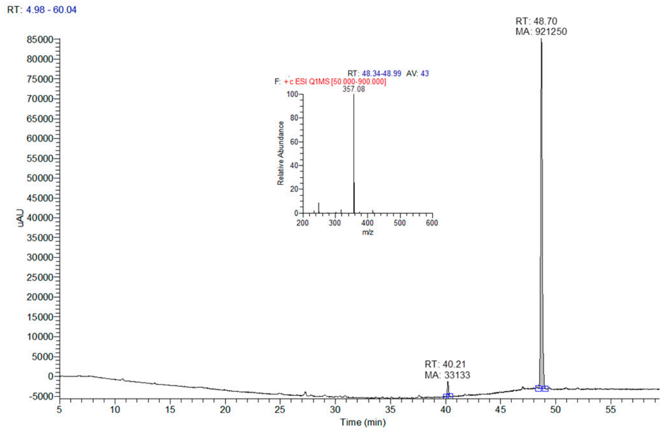

Compound 3a, UV-230 nm

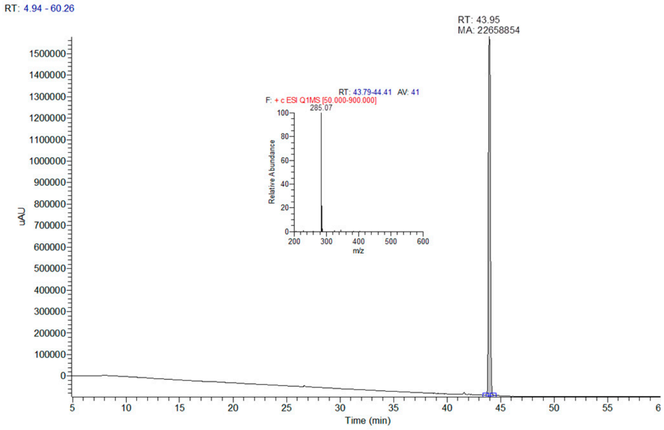

Compound 3b, UV-235 nm

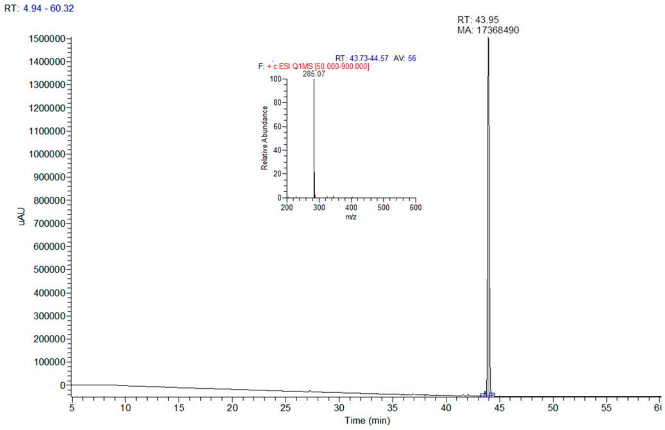

Compound 3c, UV-235 nm

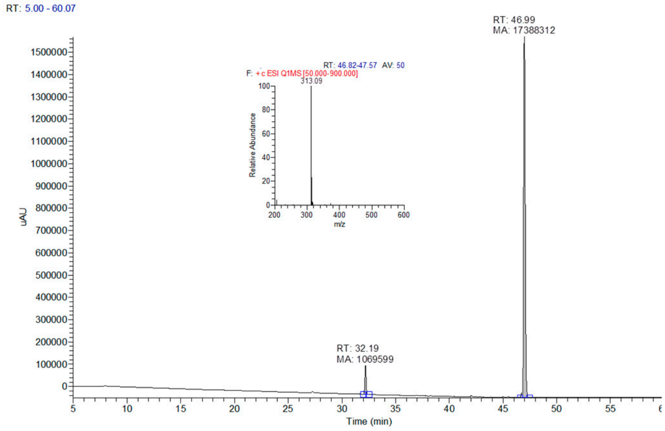

Compound 3d, UV-237 nm

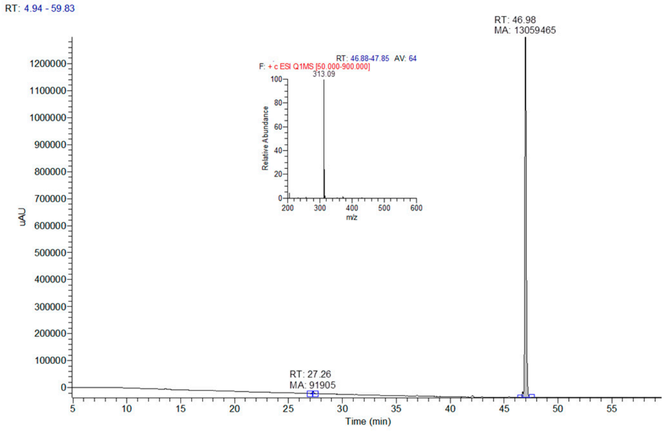

Compound 3e, UV-235 nm

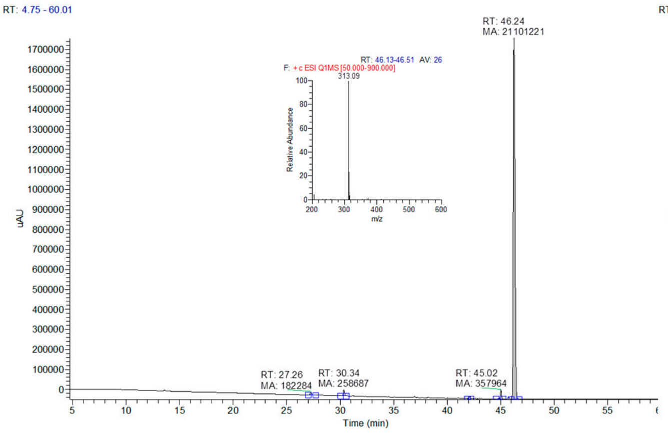

Compound 3f, UV-235 nm

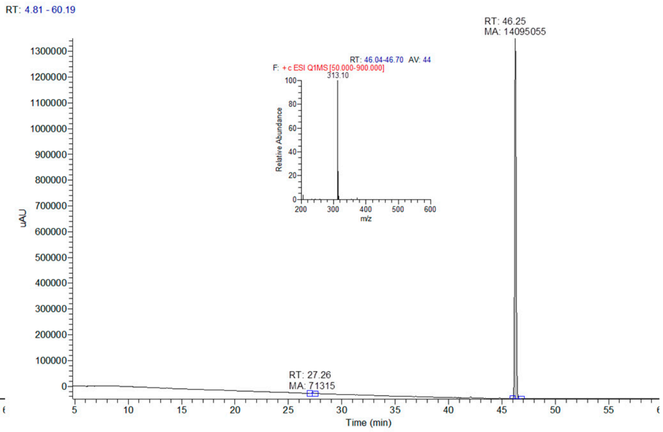

Compound 3g, UV-235 nm

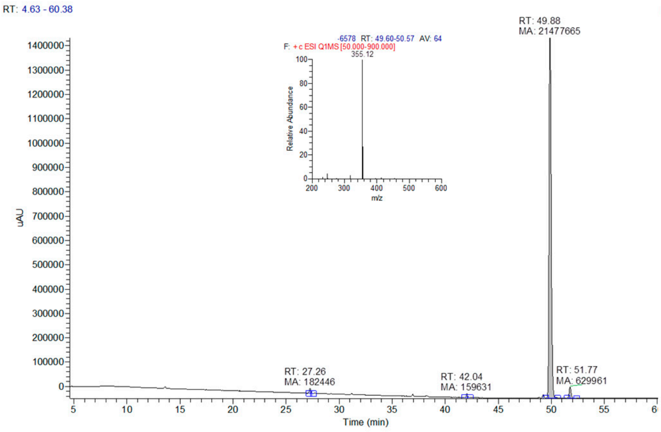

Compound 3h, UV-230 nm

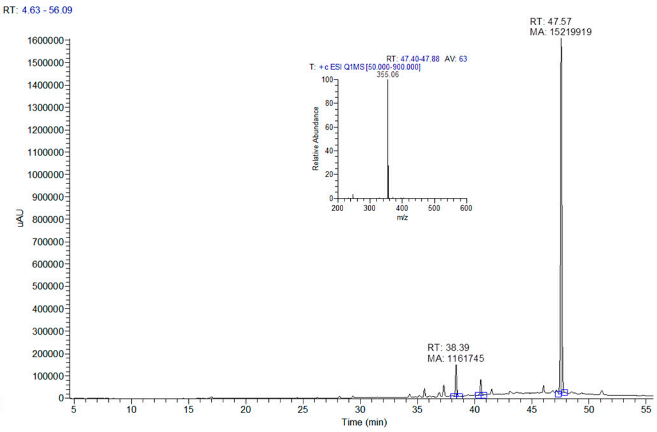

Compound 4a, UV-232 nm

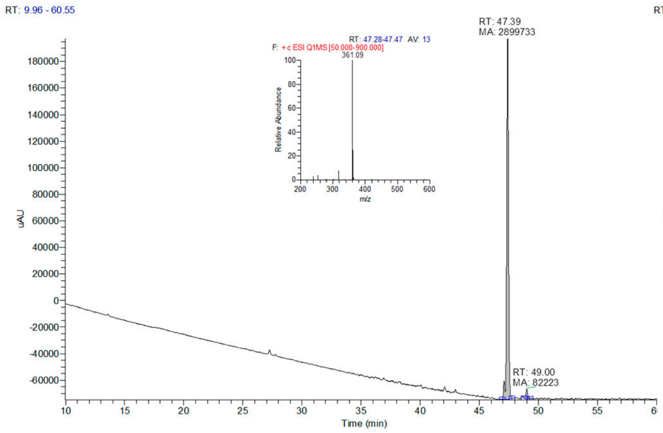

Compound 4b, UV=285 nm

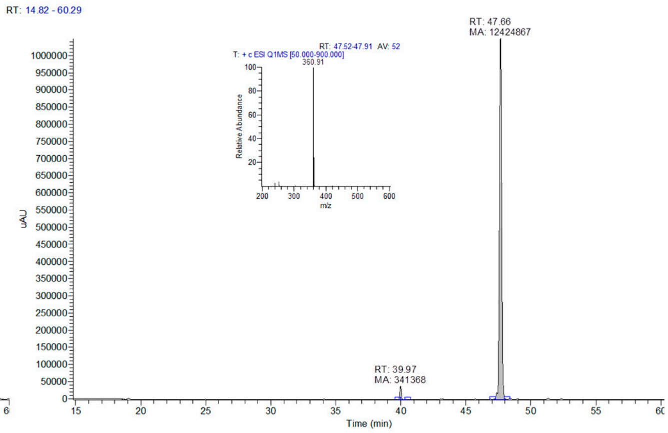

Compound 4c, UV-230 nm

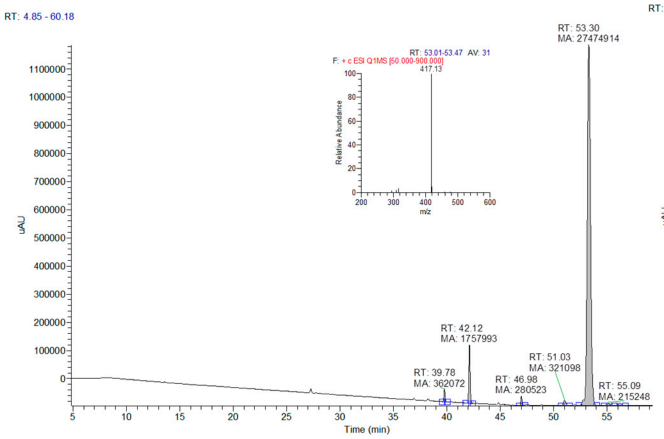

Compound 4d, UV-230 nm

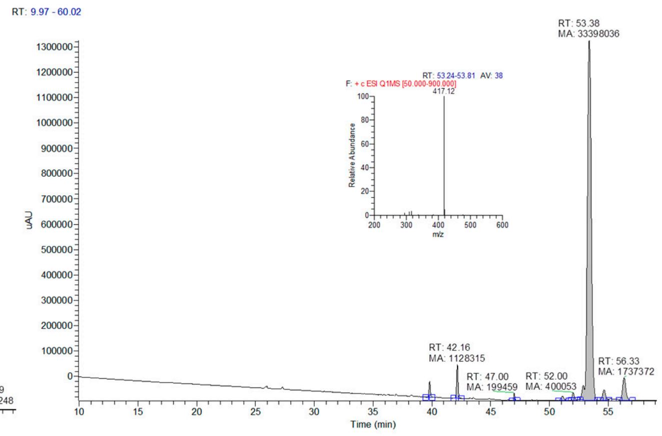

Compound 5a, UV-247 nm

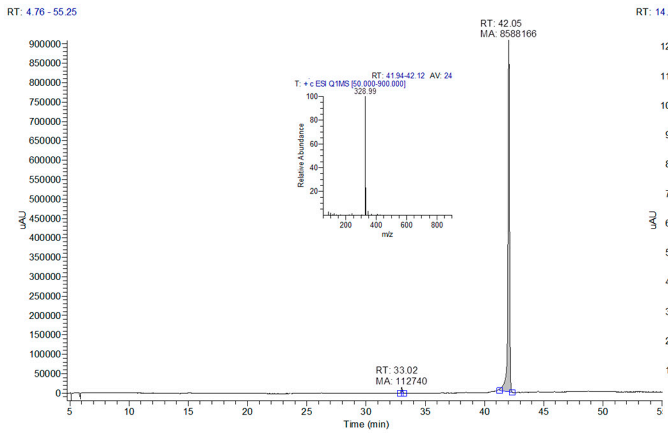

Compound 5b, UV=270 nm

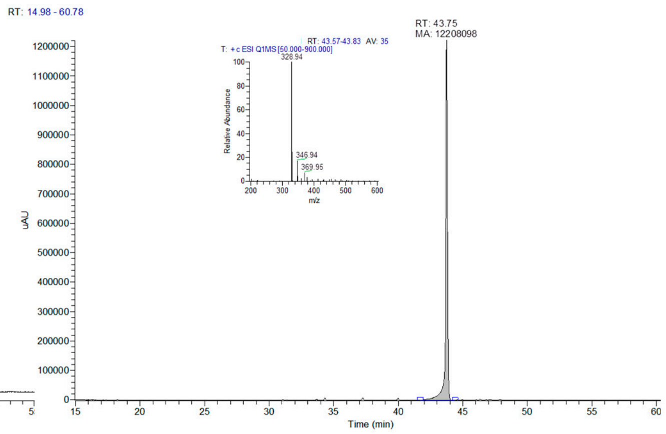

Compound 5c, UV=235 nm

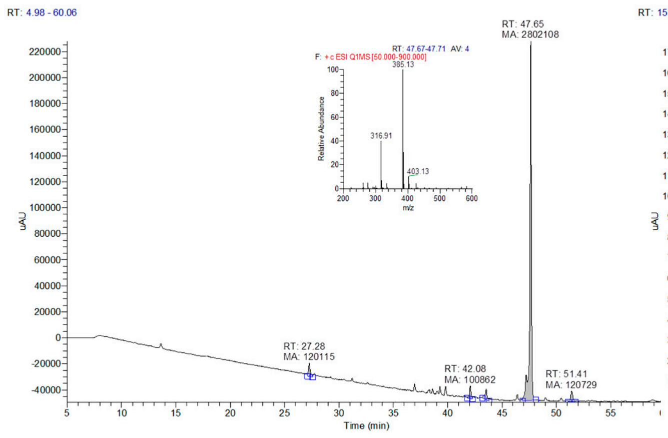

Compound 5d, UV=270 nm

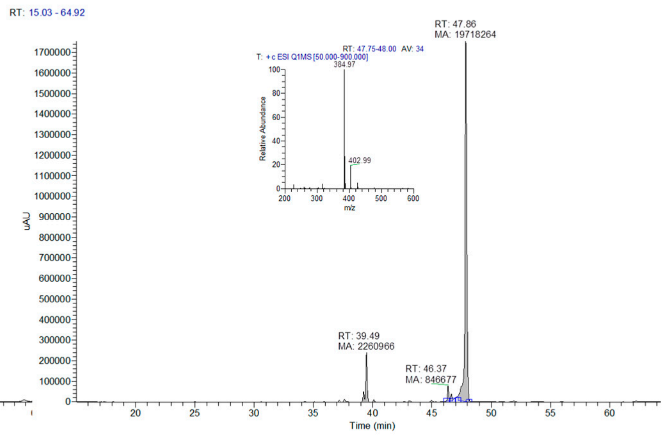

NMR data for 1b

1H-NMR-**1b**

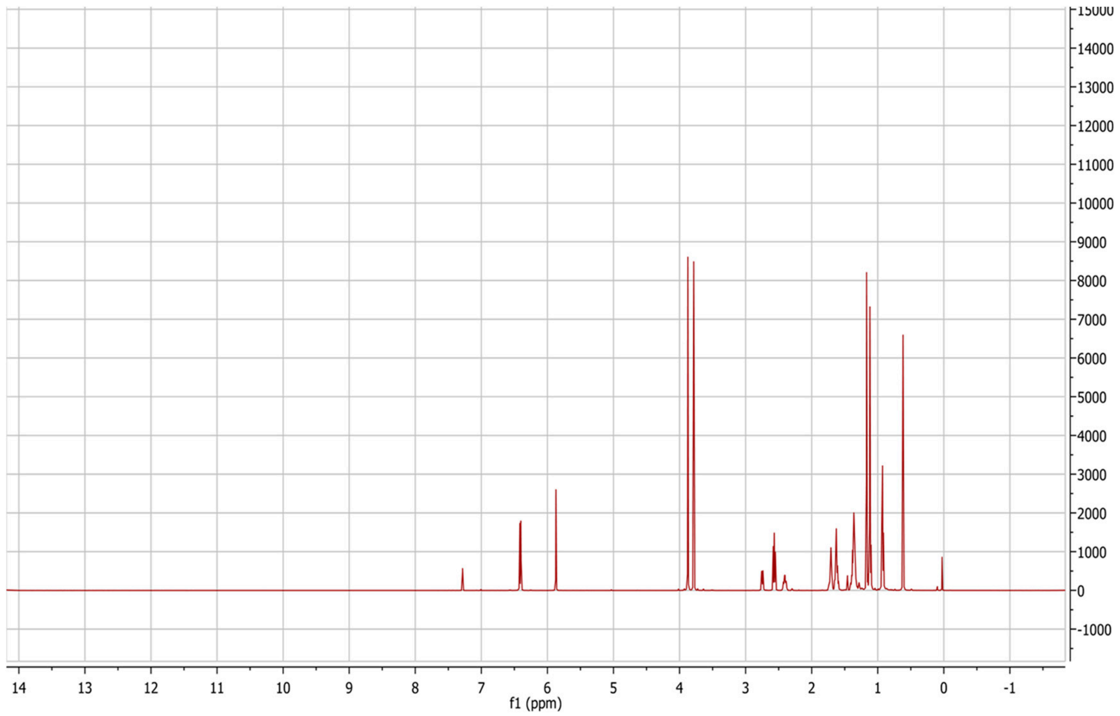

13C-NMR-**1b**

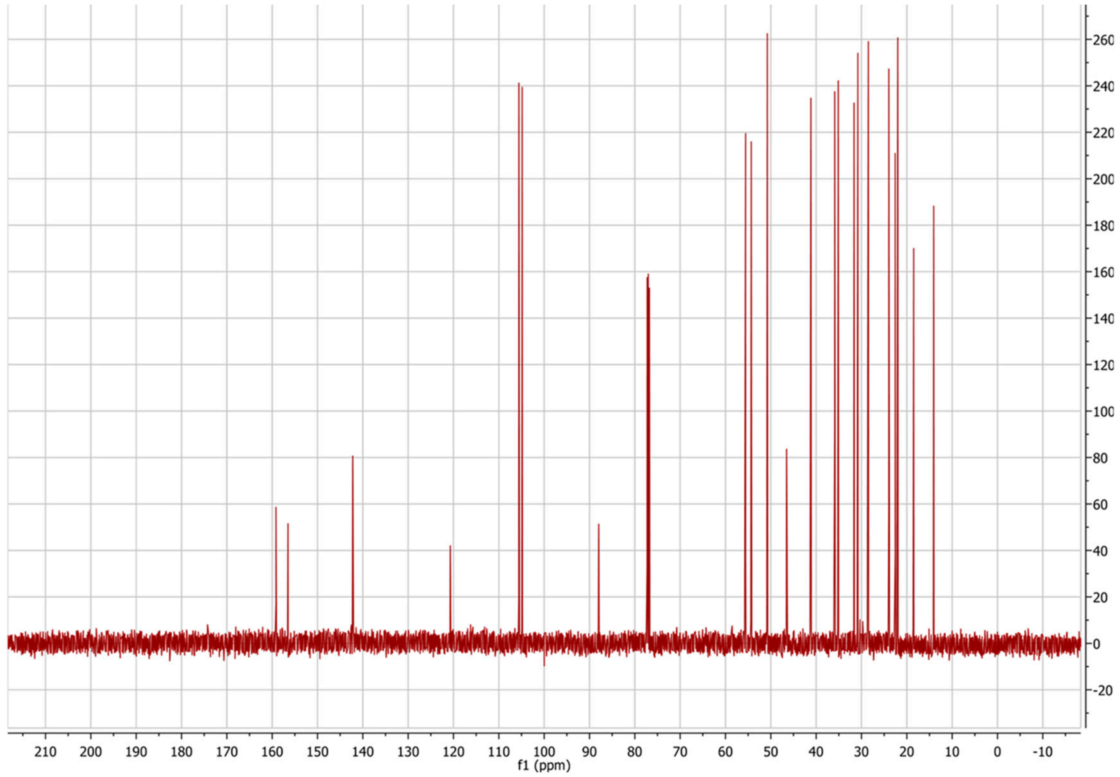

13C-DEPT-**1b**

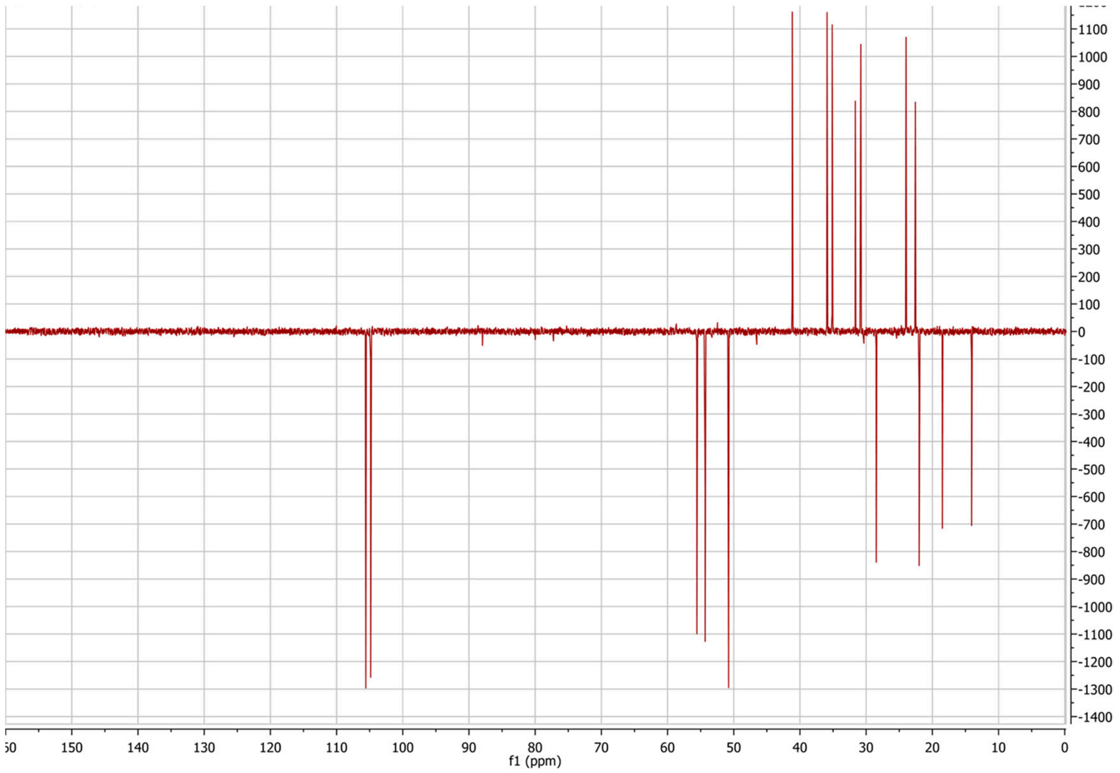

2D-HSQC-**1b**

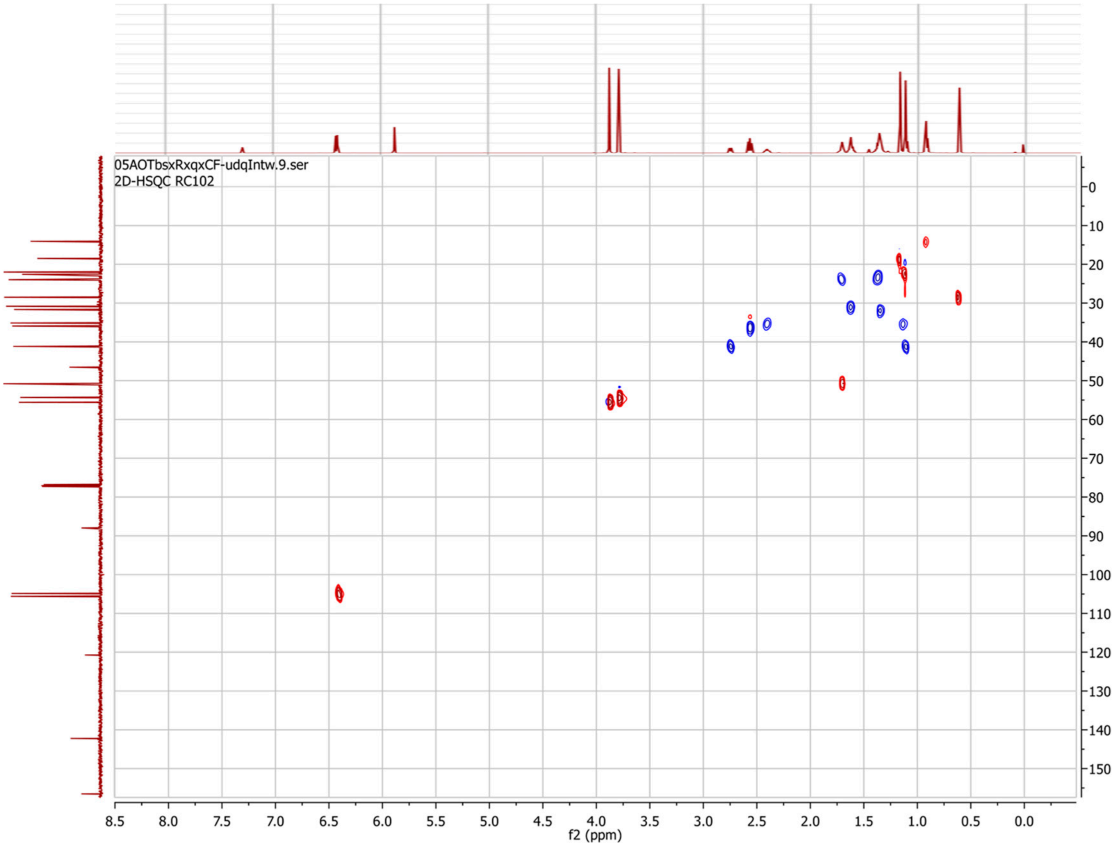

2D-HMBC-**1b**

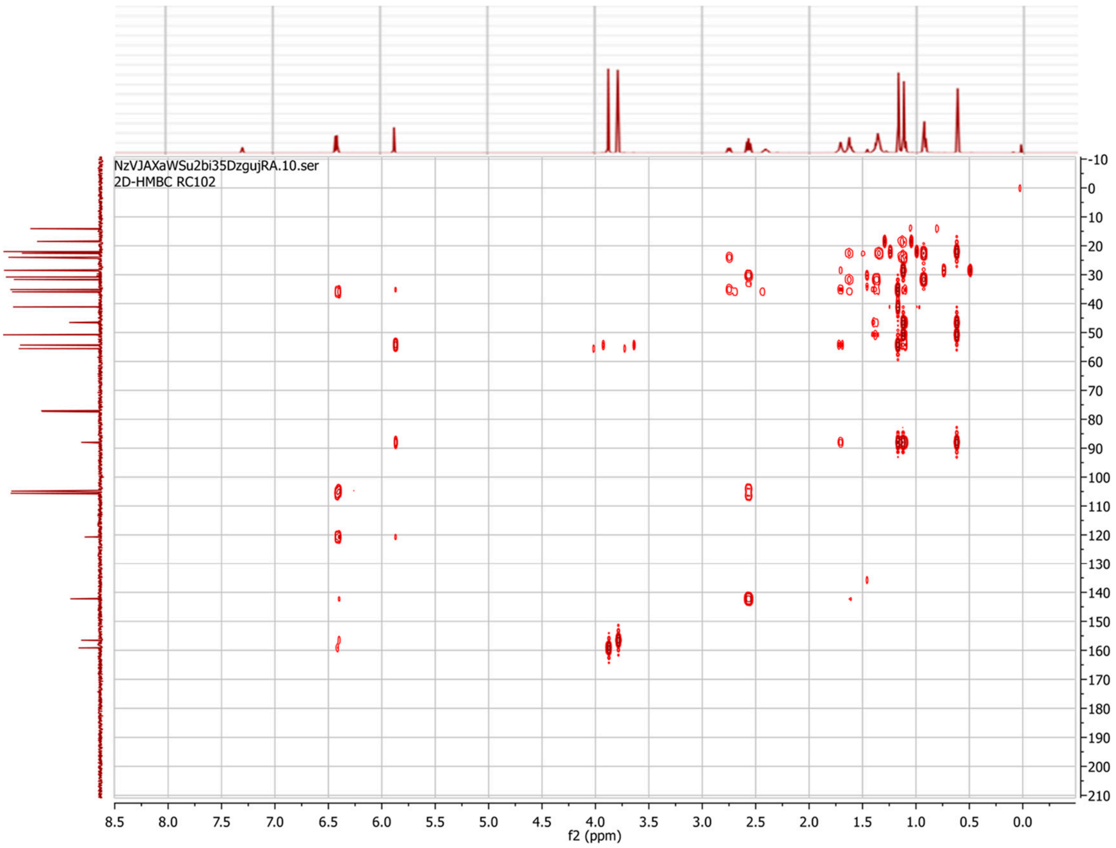

2D-COSY-**1b**

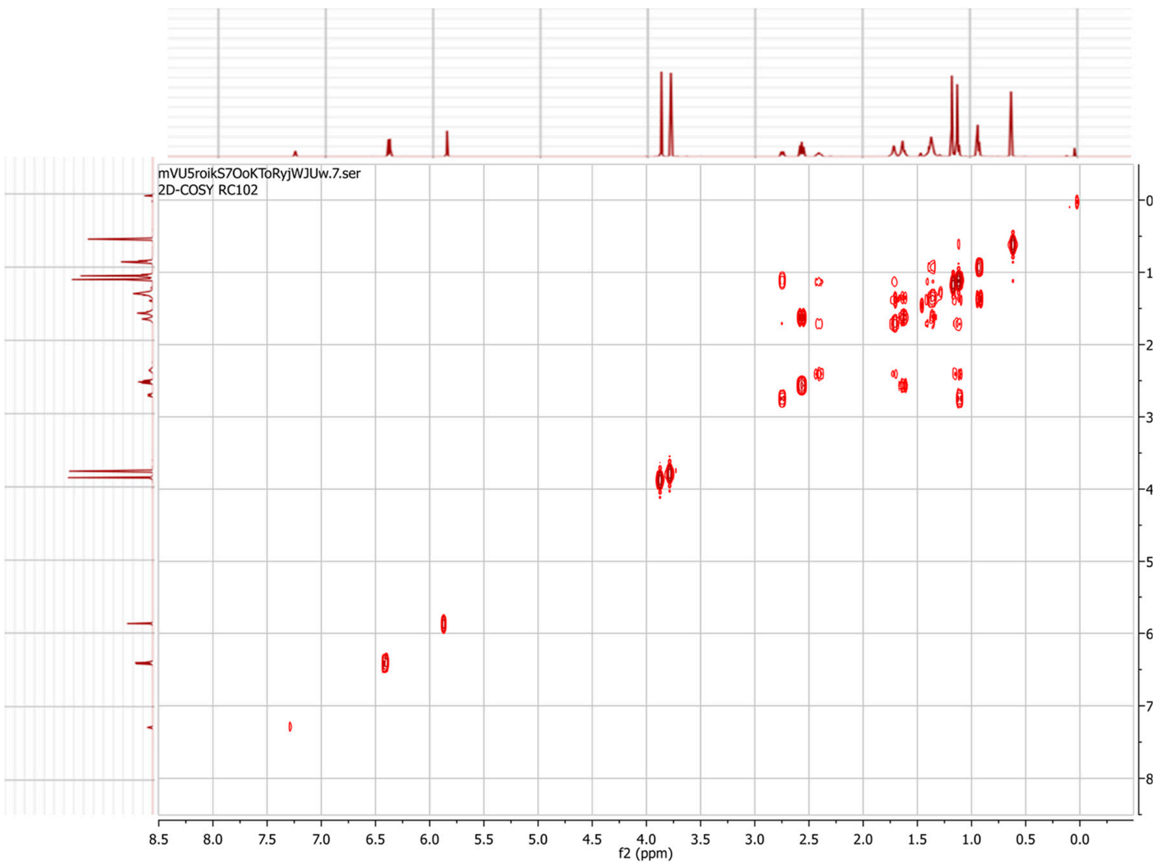

2D-TOCSY-**1b**

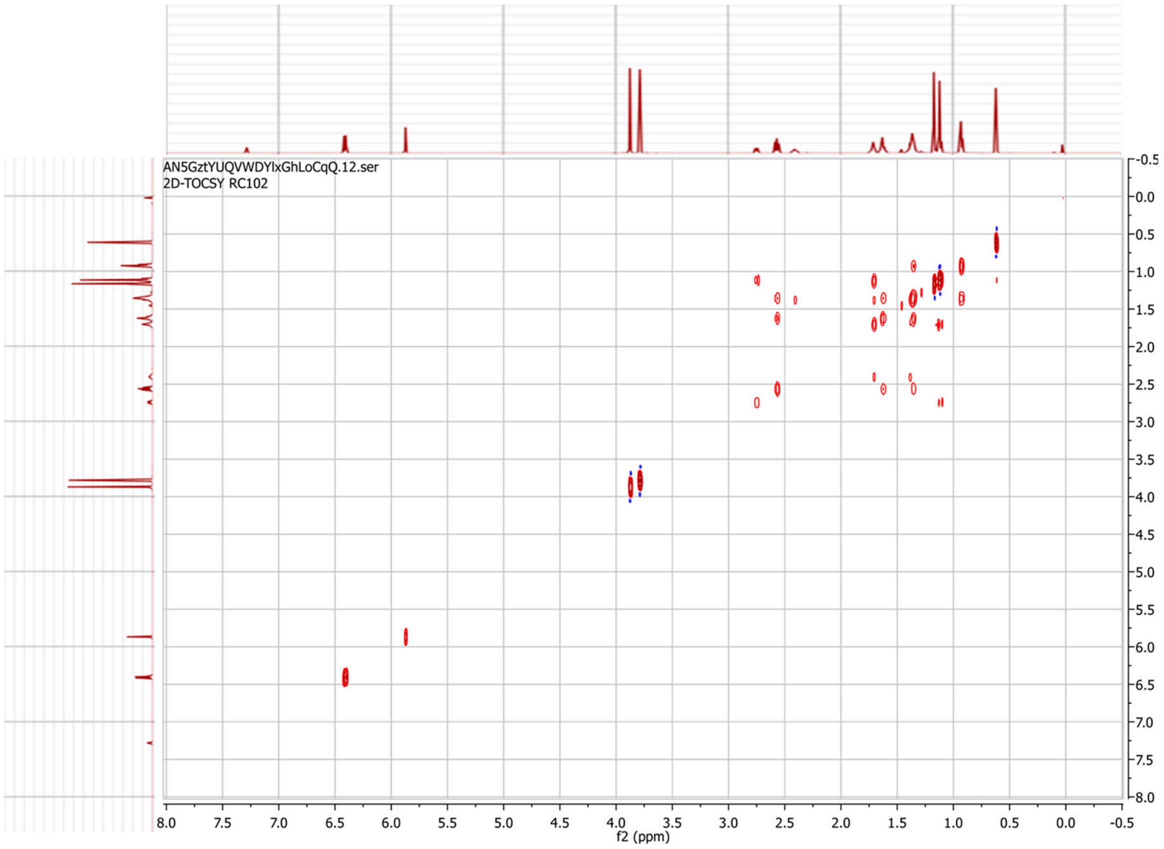

NMR data for 1d

1H-NMR-**1d**

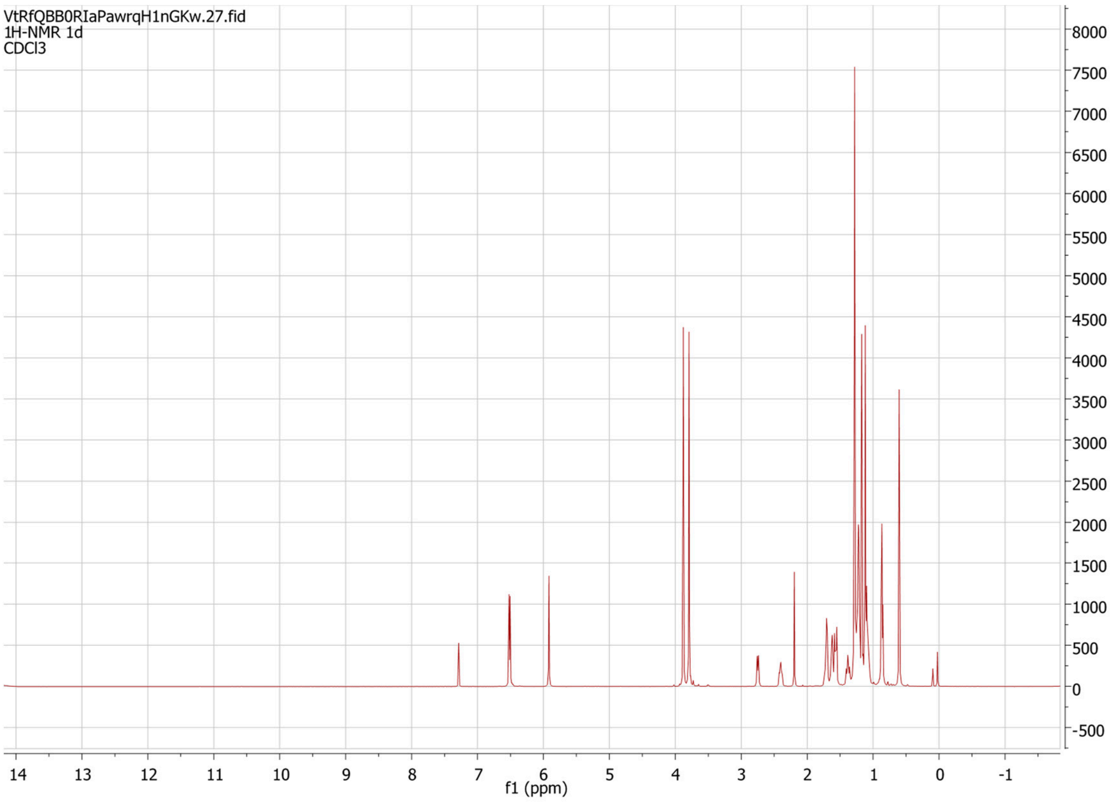

13C-NMR-**1d**

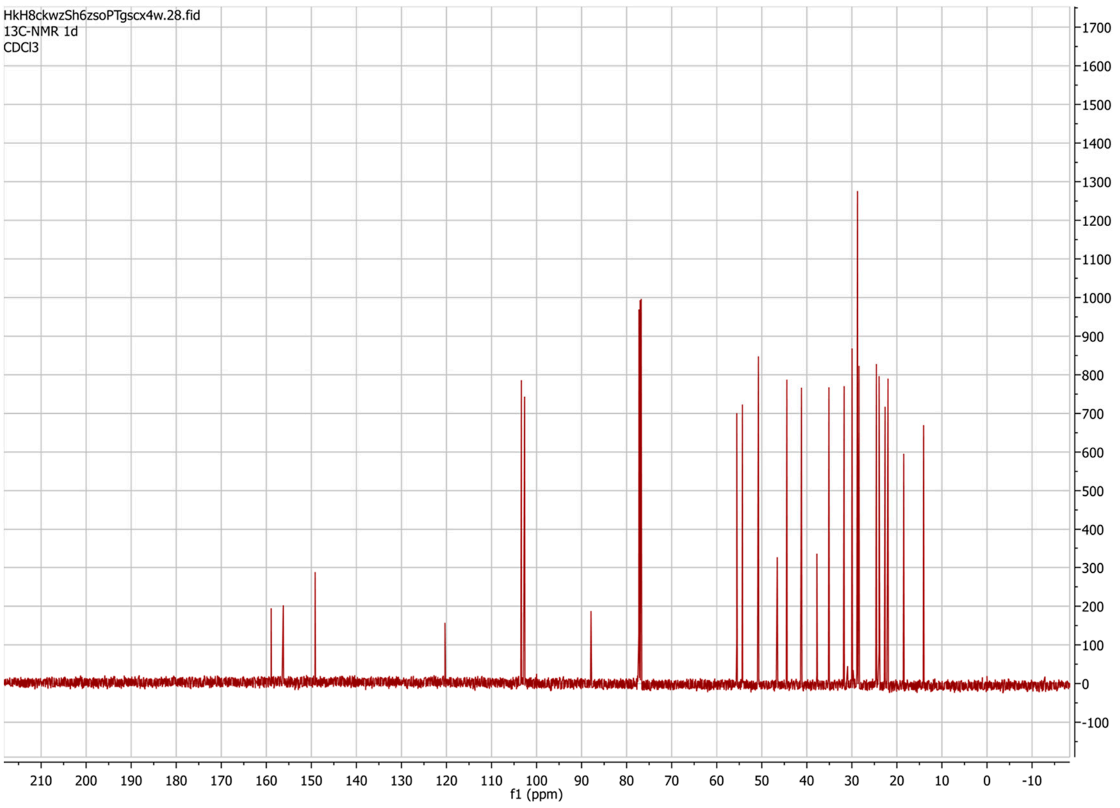

13C-DEPT-**1d**

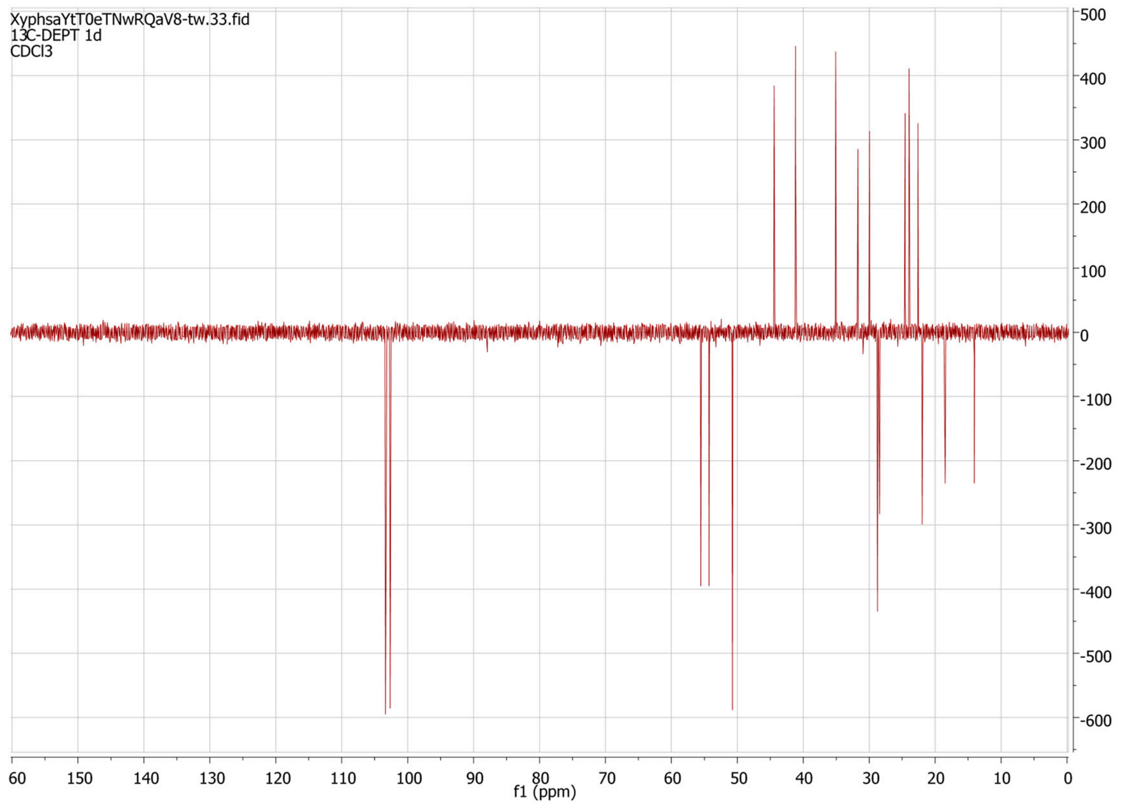

2D-HSQC-**1d**

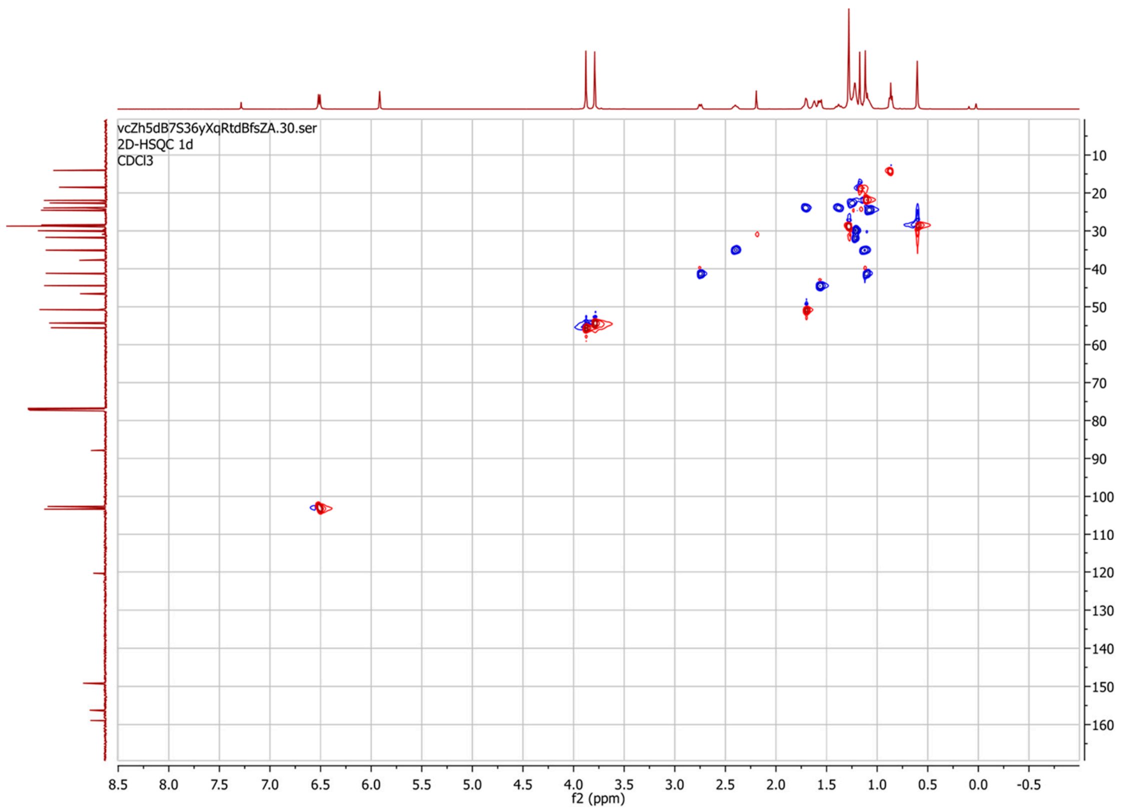

2D-HMBC-**1d**

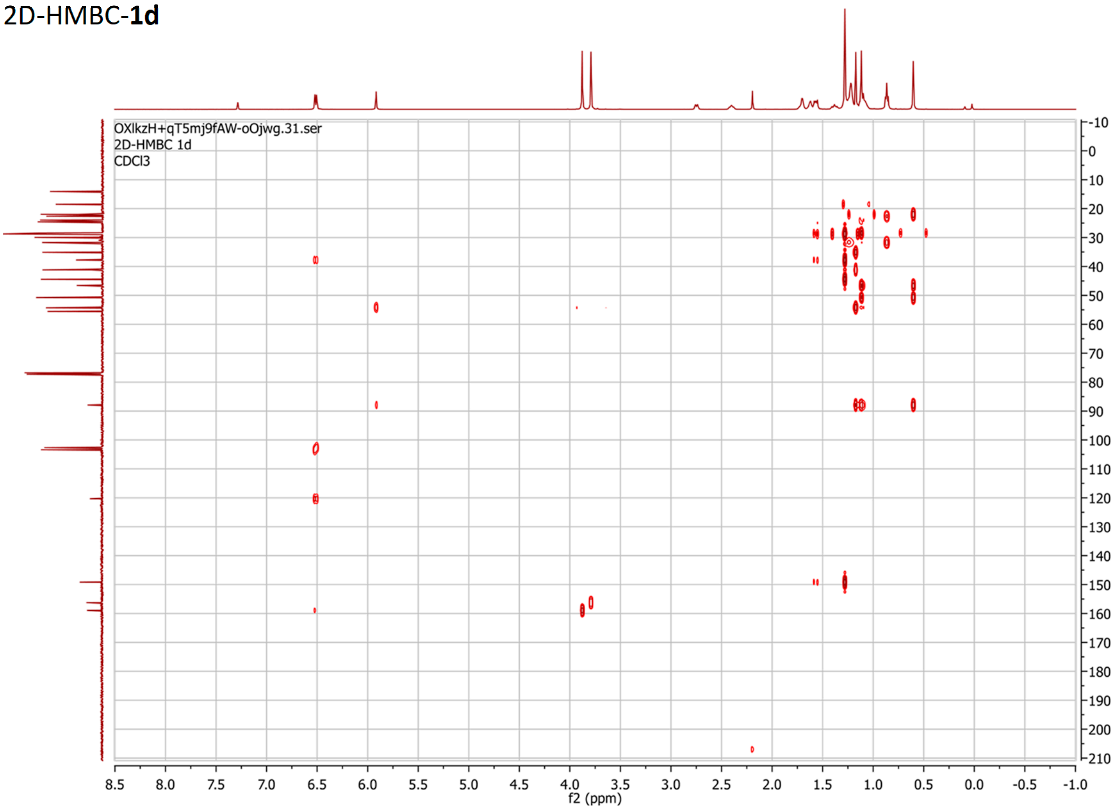

2D-COSY-**1d**

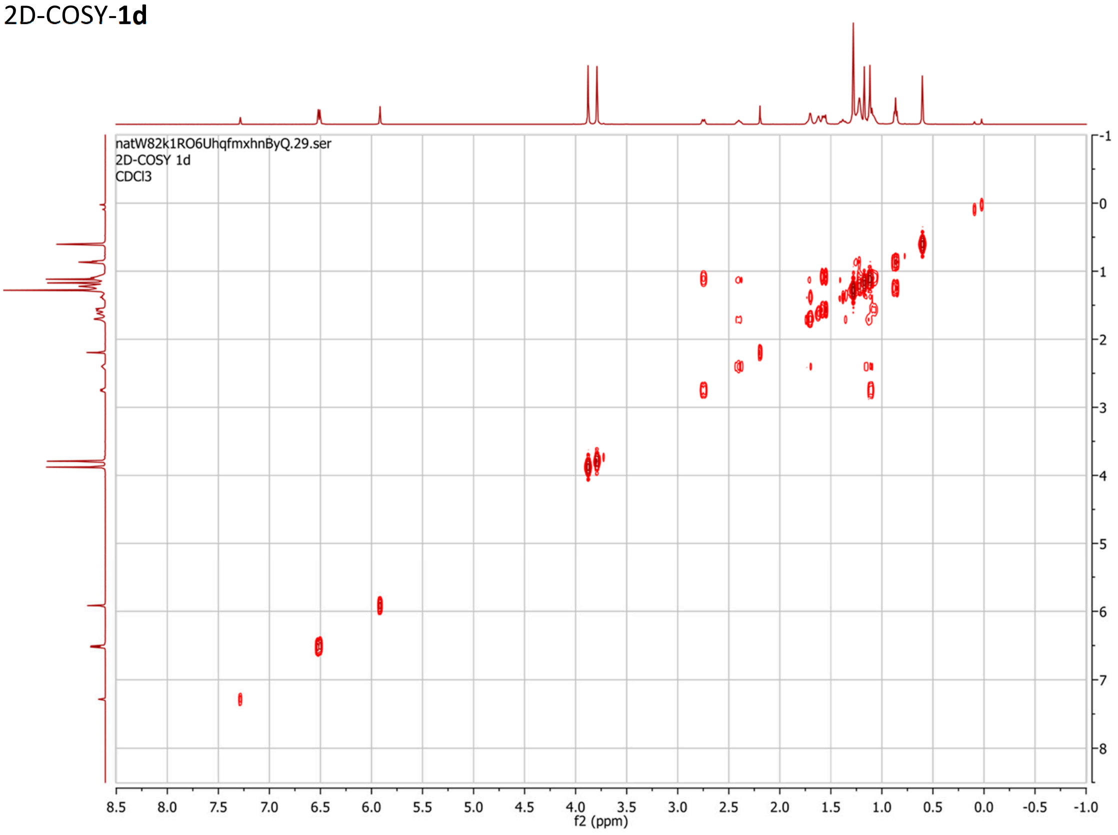

2D-TOCSY-**1d**

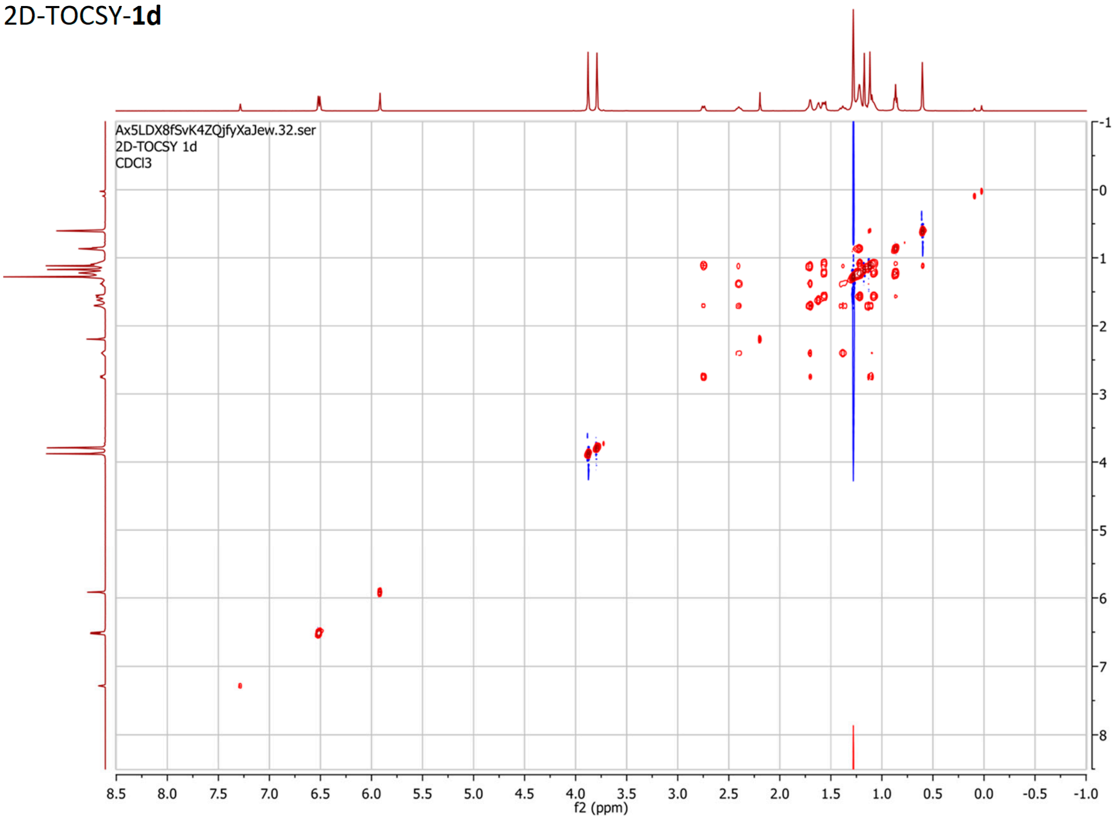
Ax5LDX8fSvK4ZQjfyXaJew.32.ser
2D-TOCSY 1d
CDCl3
8.5
8.0
7.5
7.0
6.5
6.0
5.5
5.0
4.5
4.0
3.5
3.0
2.5
2.0
1.5
1.0
0.5
0.0
-0.5
-1.0
f2 (ppm)
-1
0
1
2
3
4
5
6
7
8

NMR data for 5a

1H-NMR-**5a**

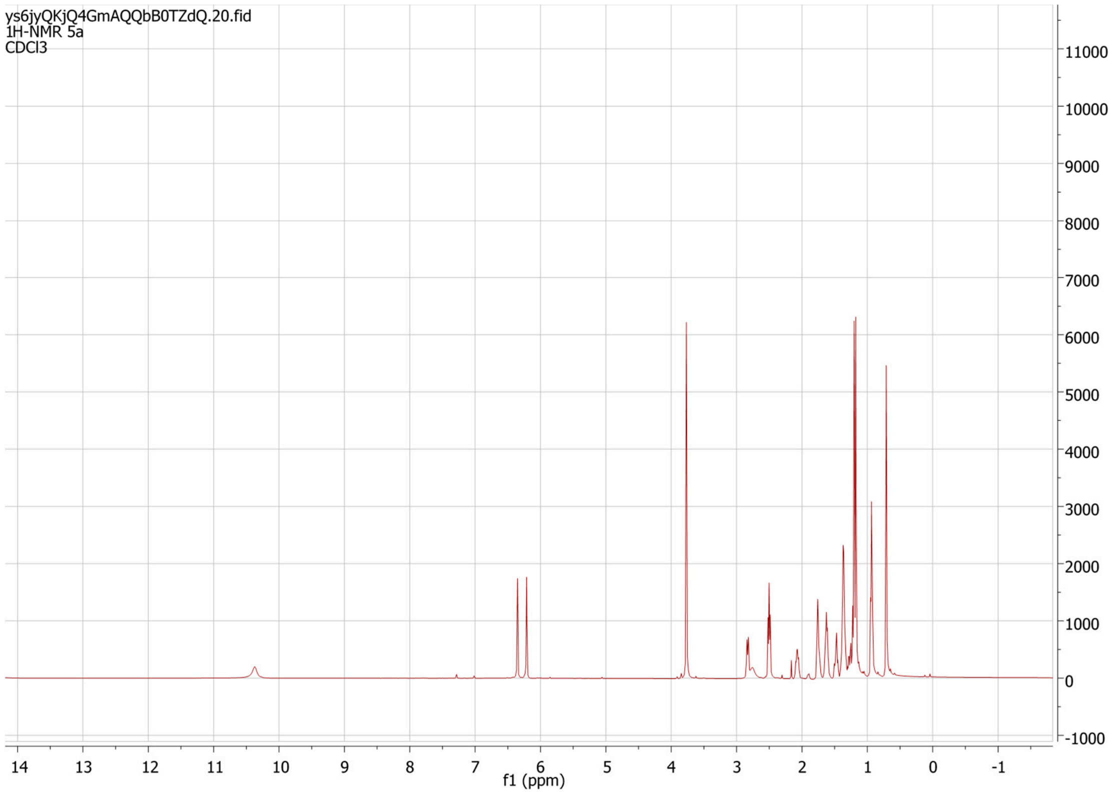

13C-NMR-**5a**

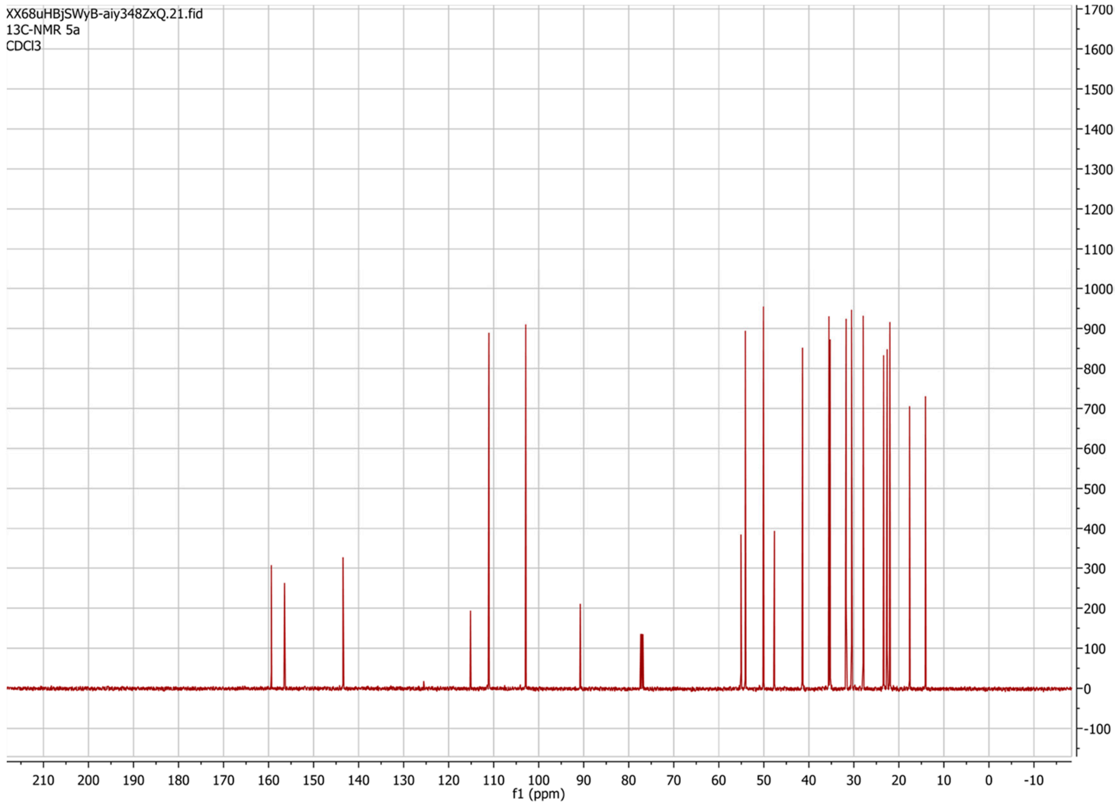

13C-DEPT-**5a**

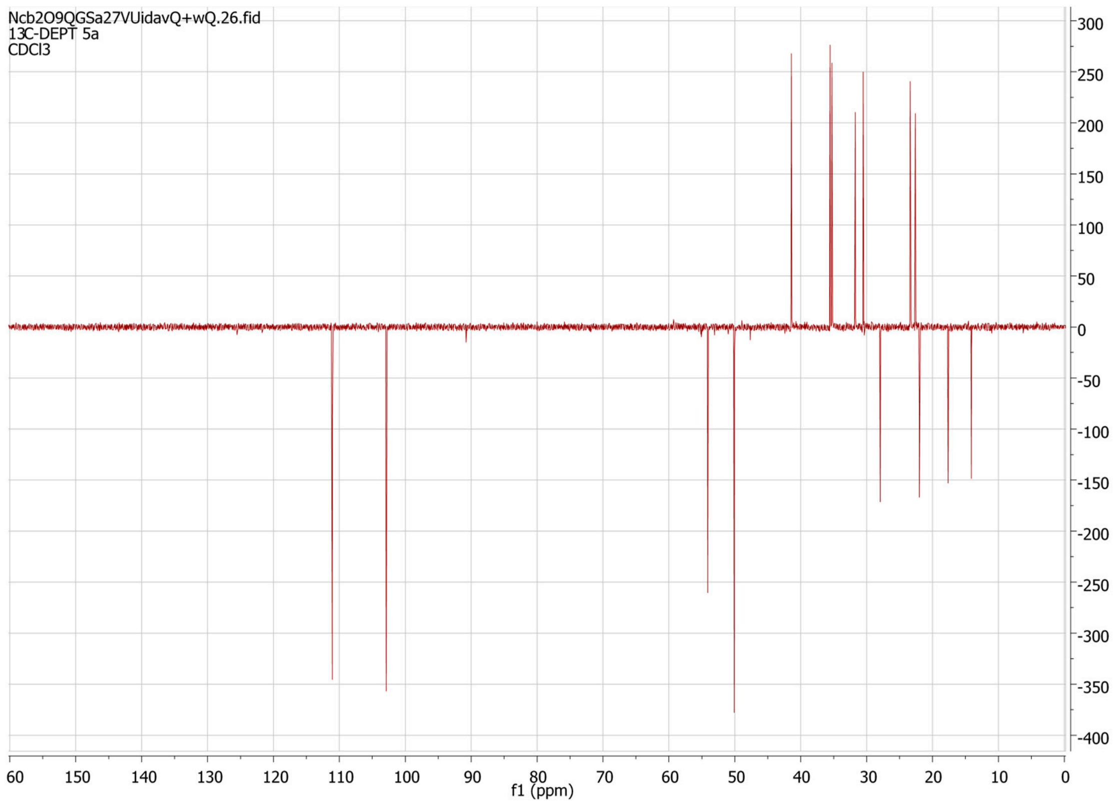

2D-HSQC-**5a**

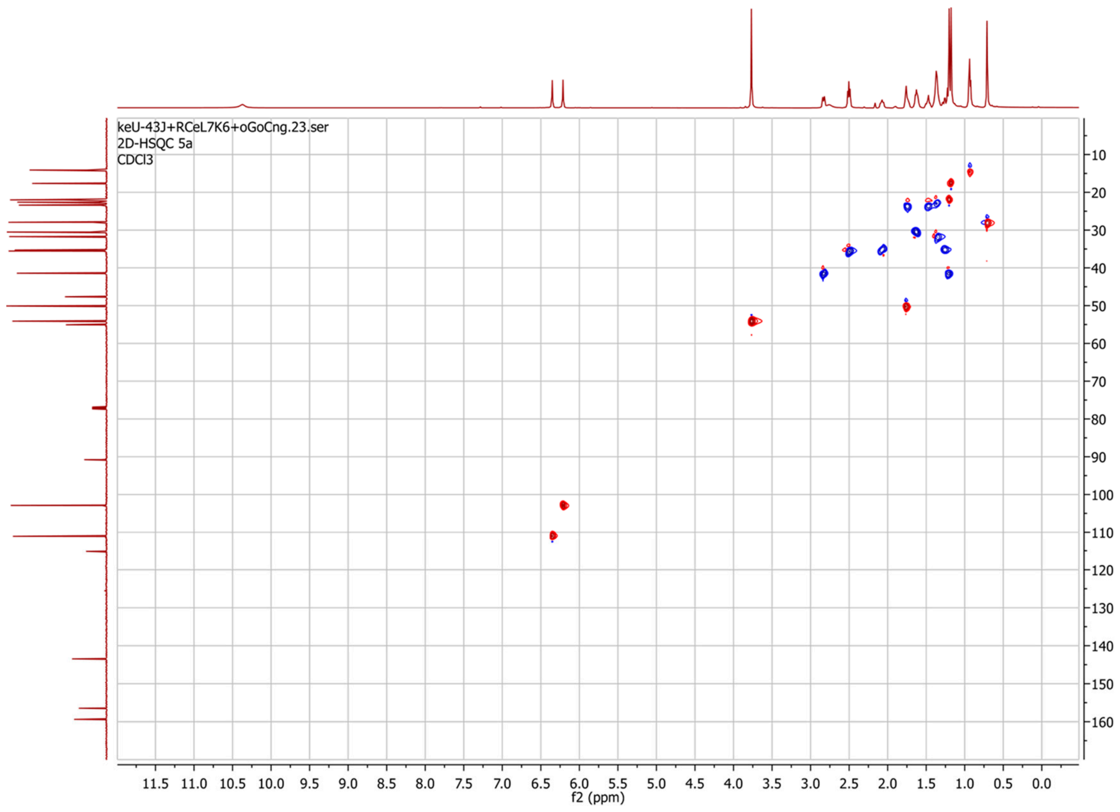

2D-HMBC-**5a**

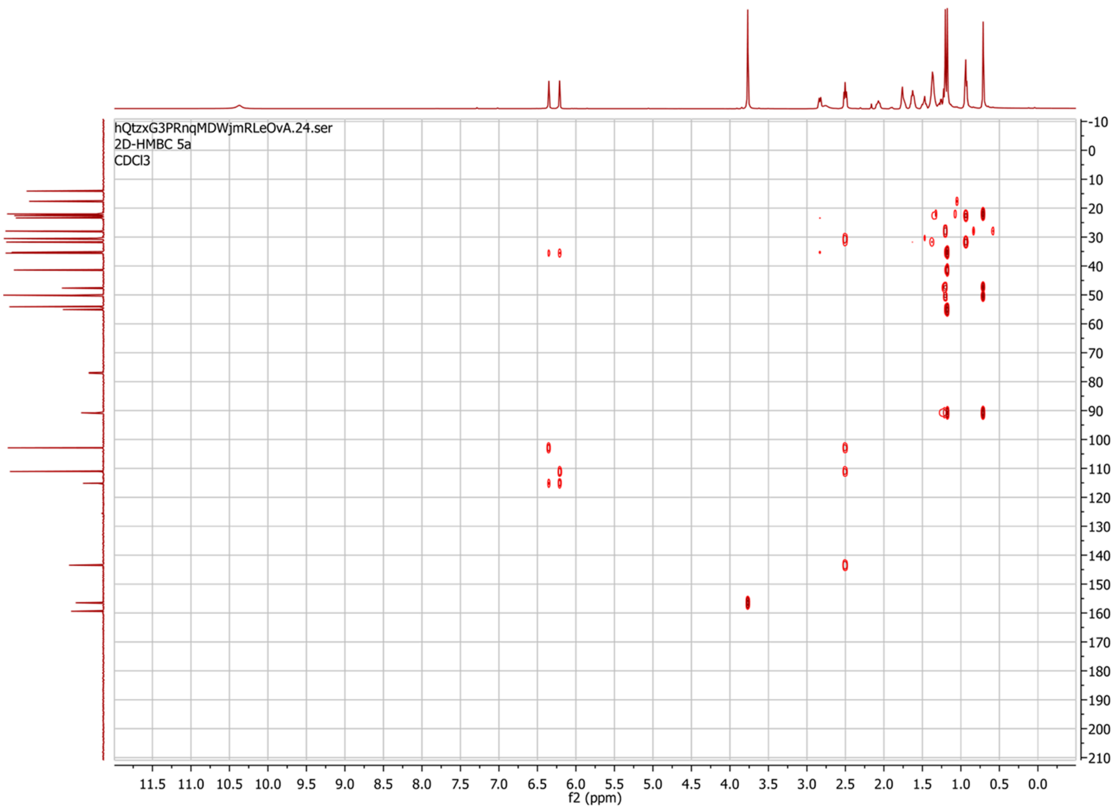

2D-COSY-**5a**

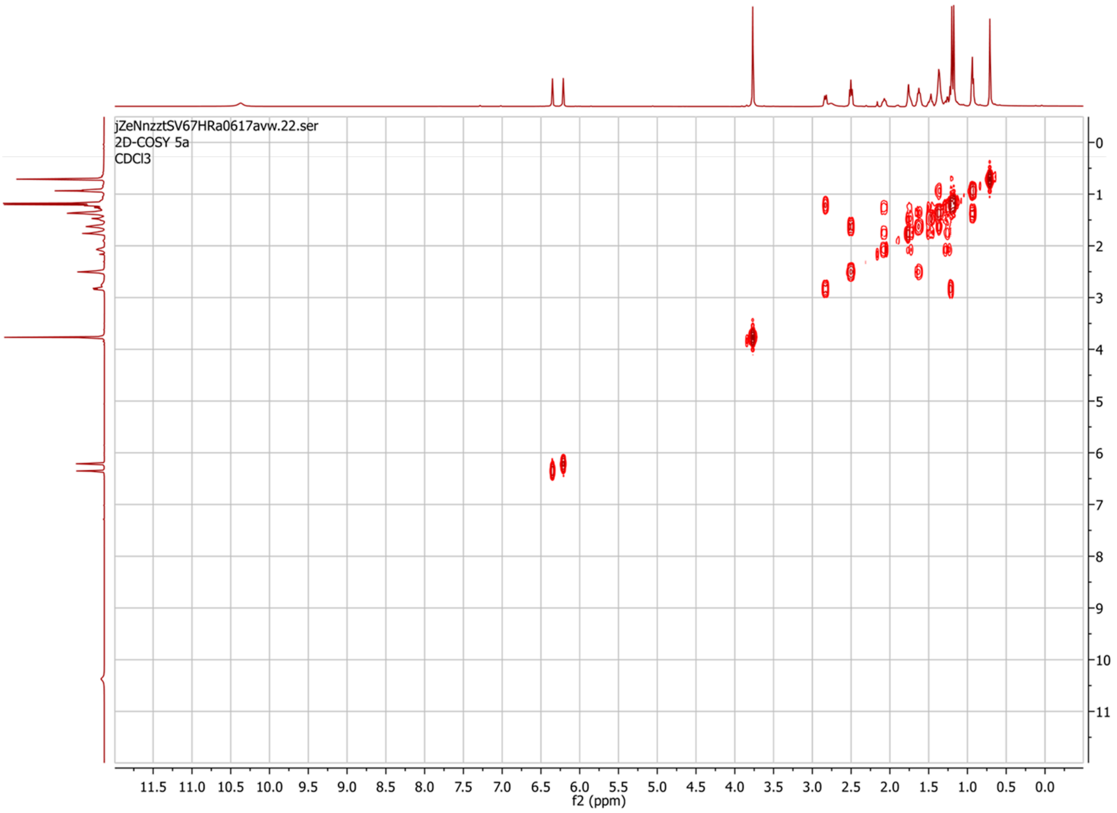

2D-TOCSY-**5a**

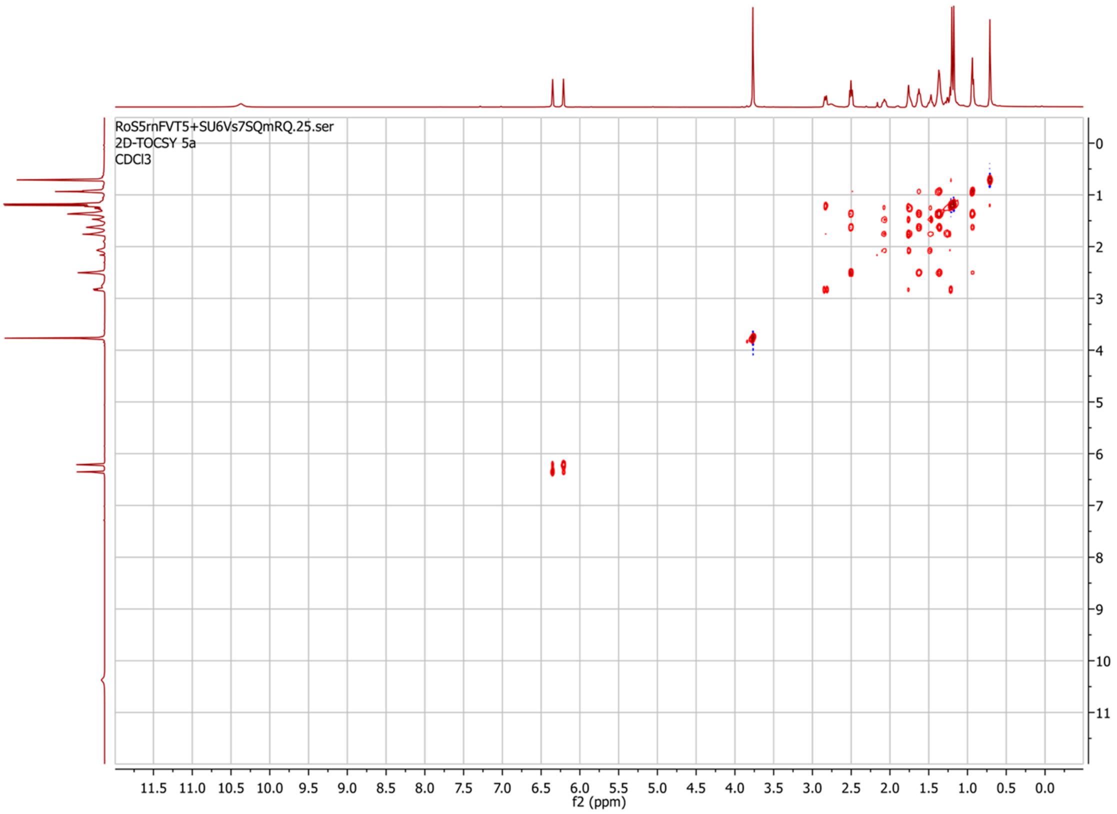
RoS5rnFVT5+SU6Vs7SQmRQ.25.ser
2D-TOCSY 5a
CDCl3
11.5 11.0 10.5 10.0 9.5 9.0 8.5 8.0 7.5 7.0 6.5 6.0 5.5 5.0 4.5 4.0 3.5 3.0 2.5 2.0 1.5 1.0 0.5 0.0
f2 (ppm)
0 1 2 3 4 5 6 7 8 9 10 11
